# Machine learning enhanced aeration systems for optimizing oxygen transfer efficiency for sustainable and safe wastewater management

**DOI:** 10.1038/s41598-025-27583-9

**Published:** 2025-12-15

**Authors:** Bishnu Kant Shukla, Arun Goel, Pushpendra Kumar Sharma, Parveen Sihag, Anoop Kumar Shukla

**Affiliations:** 1 School of Civil Engineering, Lovely Professional University, Phagwara, 144411 India; 2 Department of Civil Engineering, National Institute of Technology, Kurukshetra, 136119 India; 3 School of Civil Engineering, Lovely Professional University, Phagwara, 144411 India; 4 Department of Civil Engineering, Chandigarh University, Mohali, 140413 India; 5 Manipal School of Architecture and Planning, Manipal Academy of Higher Education, Manipal, 576104 Karnataka India

**Keywords:** Machine learning (ML), Oxygen transfer efficiency, Random forest, Artificial intelligence (AI), Environmental safety, Wastewater treatment, Environmental sciences, Civil engineering

## Abstract

This study models oxygen-transfer efficiency (OTE) in circular solid-jet aerators using a laboratory dataset of 320 observations collected under controlled conditions. Experiments varied jet count (1–8), opening area (49.24–124.03 mm²), jet length (170–470 mm), and discharge (1.05–3.04 l s⁻¹); dissolved oxygen was measured, and OTE was computed and standardized to 20 °C. Five regressors—Linear Regression (LR), M5P, Random Tree (RT), Reduced Error Pruning (REP) Tree, and Random Forest (RF)—were trained with a 70/30 train–test split and evaluated using CC, RMSE, MAE, NSE, and SI. Residual histograms with kernel-density overlays and an uncertainty summary (U95, bounds) indicated compact, slightly negative-centered errors for the tree-based models and broader, heavy-tailed errors for LR; a Taylor diagram and a Spearman heatmap supported these patterns. Among all models, RF achieved the highest test performance and the lowest errors, with results statistically superior to alternatives by paired t-tests on residuals (α = 0.05); the Spearman heatmap also showed the strongest concordance between RF predictions and observations, while a leave-one-input-out sensitivity analysis identified discharge (Q) as the dominant driver. Taken together, the results identify RF as the most accurate and generalizable predictor across the tested operating envelope, providing a practical basis for the design and optimization of aeration systems in water and wastewater treatment.

## Introduction

Water pollution continues to be a significant worldwide problem, impacting ecosystems, public health, and economic progress^1^. Aeration is a crucial component of water and wastewater treatment strategies that effectively handle the challenge of organic pollutant degradation by enriching the oxygen levels, hence improving the biological degradation process^2^. Aeration is a crucial process in both natural and constructed water systems, when air is added to water to enhance its oxygen levels. The efficacy of aeration procedures has a direct impact on the effectiveness of water treatment facilities, making it a crucial area of study and advancement^3^.

The ease and effectiveness of surface aerators in small-scale applications have made them renowned in the past. By stirring the water surface, these devices increase oxygen absorption from the air. Research executed by of Novak^4^ and Kalinske^5^ on the operational principles and efficiency of surface aerators demonstrated their usefulness in many small-scale applications of water treatment. Deeper waters experience a decrease in the efficiency of surface-based aerators, which leads to heightened energy usage and operational expenses. The limitations of surface aerators led engineers to design submerged turbine dispersers. Underwater turbines are used to diffuse air and generate small bubbles that rise up the water column in these systems. This procedure enhances the space between the skin and the ejaculation vessel for oxygen transfer. Chanson and Brattberg^6^ demonstrated that turbine dispersers placed beneath the surface can achieve higher oxygen transfer rates than surface aerators, particularly in deeper water bodies. In spite of this, these systems face challenges such as the interference from diffusers and requiring frequent servicing to maintain functionality^7,8^.

Aeration technology has evolved to include devices like liquid jet aerators that can plunge, which are becoming more sophisticated^9,10^. Aerators are used to project high-speed streams of water into the atmosphere, which then return to the water body, resulting in air being incorporated into it and a region of mixing being created. Small bubbles forming and the gas exchange interface being enlarged are responsible for a significant increase in efficiency during oxygen transfer using this technique. The operational advantages of plunging jet aerators were highlighted by Tojo and Miyanami^11^, which included their straightforward design, ease of construction, and less complicated operation in comparison to traditional systems. Deswal and Verma^12^ pointed out the effectiveness of plummeting jet aerators in large-scale applications, noting their higher energy efficiency and lower maintenance requirements. The modification of throat diameter and divergence angles by Pillai et al.^13^ greatly assisted in comprehending various aerator types, including venturi and abrupt expansion designs. They also found useful information about the design of aerators to improve their efficiency. Geometric alterations in the performance of aerators were investigated by Pasrija and Pillai^14^. These devices’ design was improved, leading to increased efficiency as a result of to their extensive findings.

In recent years, incorporating artificial intelligence (AI) and soft computing methods into environmental engineering has greatly enhanced the optimisation and performance of aeration systems. These advanced methods enable the analysis of complex, extensive datasets to identify optimal operational conditions and design parameters. M5P Tree, Random Forest (RF), a simpler version of the traditional statistical methods, as well as other soft computing techniques like Linear Regression (LR), Reduced Error Pruning (REP) Tree and Random Tree (RT) have been utilized to optimize the design and operational parameters of aeration systems. Energy efficiency and the performance of systems have seen considerable advancements through these methodologies^15,16^. Recent lab-to-field work on stepwise cascade aeration showed gradient boosting as the most accurate aeration efficiency predictor (coefficient of determination, R² = 0.96; mean absolute error, MAE = 0.027), enabling geometry–flow optimisation and successful full-scale validation^17^. For biological aeration, PSO-based optimisation of HRT reduced blower energy by approximately 37.6%, with airflow rate and temperature identified as the dominant levers^18^. The M5P Tree approach has been utilized to model and predict aeration systems by adding linear functions at the terminal nodes, which is an extension of regression trees. The M5P model tree was introduced by Quinlan^19^ as a practical method to manage continuous attributes and create accurate models that can be pruned to avoid overfitting. In Venturi flumes, a head-to-head comparison (MLR/MNLR, GBM/XGB, RF, M5, RT, REP) found unpruned M5P top-performing (correlation coefficient, CC = 0.946; lowest errors), with throat width and downstream gauge as key controls from sensitivity/SHAP^20^. Environmental engineering applications frequently make use of this technique to depict intricate interactions. Random Forest technique, introduced by Breiman^21^, is a widely used method for managing nonlinear and complex relationships in large datasets. A Random Forest is a collection of decision trees that improves system dynamics and enhances prediction accuracy. Studies conducted by Chen et al.^22^ demonstrated the feasibility of using Random Forests for the optimization of aeration systems in various engineering applications. Several REP Tree techniques have been utilized to optimize aeration operations, as they are known for their ability to generate thousands of repeats and select the most suitable tree by considering variation and information gain. The process of the REP Tree technique, as described by Witten et al.^23^, involves reducing error rates and improving the model’s dependability by eliminating less efficient branches. The use of this technique is particularly advantageous in environmental engineering, where accurate forecasting is crucial for system optimization. Using Random Tree techniques, which involve selecting a random subset of data to construct decision trees, can be used to model complex ponds and towers without the need for extensive pruning. According to Sürer et al.^24^, the use of Random Trees was found to be beneficial in producing well-balanced trees that make predictions more comprehensible and more accurate. These algorithms are capable of handling both classification and regression problems, making them versatile tools in aeration technology. LR, although less complex, is still useful in building early models for improving aeration efficiency. Linear regression (LR) establishes a direct correlation between oxygen transfer efficiency (OTE) and the input variables. It provides a framework for developing more complex models and serves as an indicator of the efficiency or performance of sophisticated soft computing techniques^25^.

A significant shift in environmental engineering is being brought about by the integration of artificial intelligence (AI) and soft computing approaches into aeration technology. A constraint-aware inverse-support vector machine (SVM) controller for SBRs dynamically tuned aeration to keep effluent NH₃-*N* < 5 mg/L while cutting energy by 20%, outperforming fixed-rate/PID schemes^26^. Kumar et al.^27^ conducted a thorough analysis using multiple nonlinear regression (MNLR), artificial neural network (ANN), adaptive neuro-fuzzy inference system (ANFIS), multivariate adaptive regression splines (MARS) and generalized regression neural network (GRNN) techniques to predict the efficiency of oxygen transfer in hollow jet aerators with variable apertures. Their research demonstrated that the ANFIS and ANN strategies were most accurately predicted, while MNLR, MARS, and GRNN methods followed closely behind. The study highlighted the significance of the jet velocity as a crucial input variable, and the sensitivity analysis demonstrated its substantial influence on oxygen transfer coefficient. Across effluent-quality prediction (COD, BOD, TSS, N, P), ensemble models (XGBoost/GB/LightGBM/RF) consistently outperformed DT baselines, with VSS ranked most informative across SelectKBest, MI, and RF-RFE^28^. In his influential work on Random Forests, Breiman^29^ demonstrated that it is capable of handling relatively large datasets and complex relationships, making it a suitable tool for environmental engineering. A multimodal machine learning (ML) framework (RF with visual signals) achieved MAE = 4.4 and R² = 0.95 for aeration forecasting and lowered full-scale operating costs by 19.8%, with open-source release supporting adoption^30^. By developing the M5P Tree, Quinlan^19^ introduced a robust structure for modeling continuous attributes, which has since been widely applied to improve accuracy and operational efficiency in related research. Baylar et al.^31^ analyzed various aerator designs, including venturi and abrupt expansion types, under different geometric setups. Their study offered critical insights into optimizing aerators, particularly by focusing on parameters such as throat diameter and divergence angles, which significantly enhance aeration performance. Deswal and Verma^32^ examined the impact of multiple plunging jets on the efficiency of oxygen transfer. The study highlighted the importance of incorporating both jet configuration and power input to optimize aeration performance.

Despite extensive research on aeration devices, very few studies have rigorously examined the oxygen transfer efficiency (OTE) of solid jet aerators with circular orifices using advanced machine learning approaches. Previous works largely focused on geometric modifications or limited-scale empirical modelling, leaving a gap in predictive tools capable of capturing the complex, nonlinear interactions of multiple operational parameters such as discharge rate, jet length, aperture area, and number of jets. Accurate prediction of OTE is critical for improving energy efficiency, reducing treatment costs, and ensuring reliable aerator design. To address this gap, a systematic evaluation of both conventional and advanced algorithms is needed to determine which frameworks most effectively represent aeration dynamics under varied operating conditions.

Accordingly, the objective of this study is to model and predict the OTE of solid jet aerators with circular apertures by applying a spectrum of AI/ML techniques—Linear Regression (LR), M5P model tree, Random Tree (RT), Reduced Error Pruning (REP) Tree, and Random Forest (RF). These methods were chosen to span complementary strengths: LR as a conventional baseline, M5P for piecewise linear regression with interpretability, RT and REP Tree for capturing nonlinear dynamics through randomized or pruned trees, and RF as a robust ensemble approach with strong generalization ability. The models were trained and validated on a comprehensive dataset spanning jet numbers (1–8), jet lengths (170–470 mm), aperture areas (49.24–124.03 mm²), and discharges (1.05–3.04 l/s). By capturing nonlinear interactions across these operational variables, this study provides one of the earliest systematic comparisons of multiple AI/ML algorithms for solid jet aerators, thereby advancing the understanding of aeration dynamics and offering recommendations for designing and operating more efficient aeration systems.

**Study contributions.** (i) A rigorous, like-for-like comparison of LR, M5P, RT, REP Tree, and RF on a 320-record experimental dataset for circular solid-jet aerators; (ii) evidence that RF generalizes best on held-out tests (with statistical metrics, scatter plots, paired t-tests, Taylor/box plots, and correlation analysis), while RT’s stronger training scores do not persist under testing; and (iii) a transparent, reproducible workflow (flowchart + checklist) and leave-one-input-out sensitivity that identifies discharge (Q) as the dominant driver of OTE.

This study is subject to certain limitations and assumptions. The dataset was obtained under controlled laboratory conditions, which may not fully reflect large-scale or field variability. Only circular solid-jet aerators were examined within fixed ranges of discharge and geometry; extension to other aerator types and operating environments is needed for broader generalization. Experiments were conducted under variable ambient temperatures, with all oxygen-transfer results standardized to 20 °C using the temperature-correction relation; long-term effects (e.g., fouling, turbulence nonstationarity) were not modelled. These constraints frame the interpretation of results and point to future work.

Overall, combining established aeration concepts with modern soft-computing methods advances environmental engineering practice. The proposed framework offers practical guidance for selecting and tuning data-driven models to enhance aeration performance in water and wastewater treatment.

## Materials, methods and computational framework

### Experimental configuration and setup

**Fig. 1:**
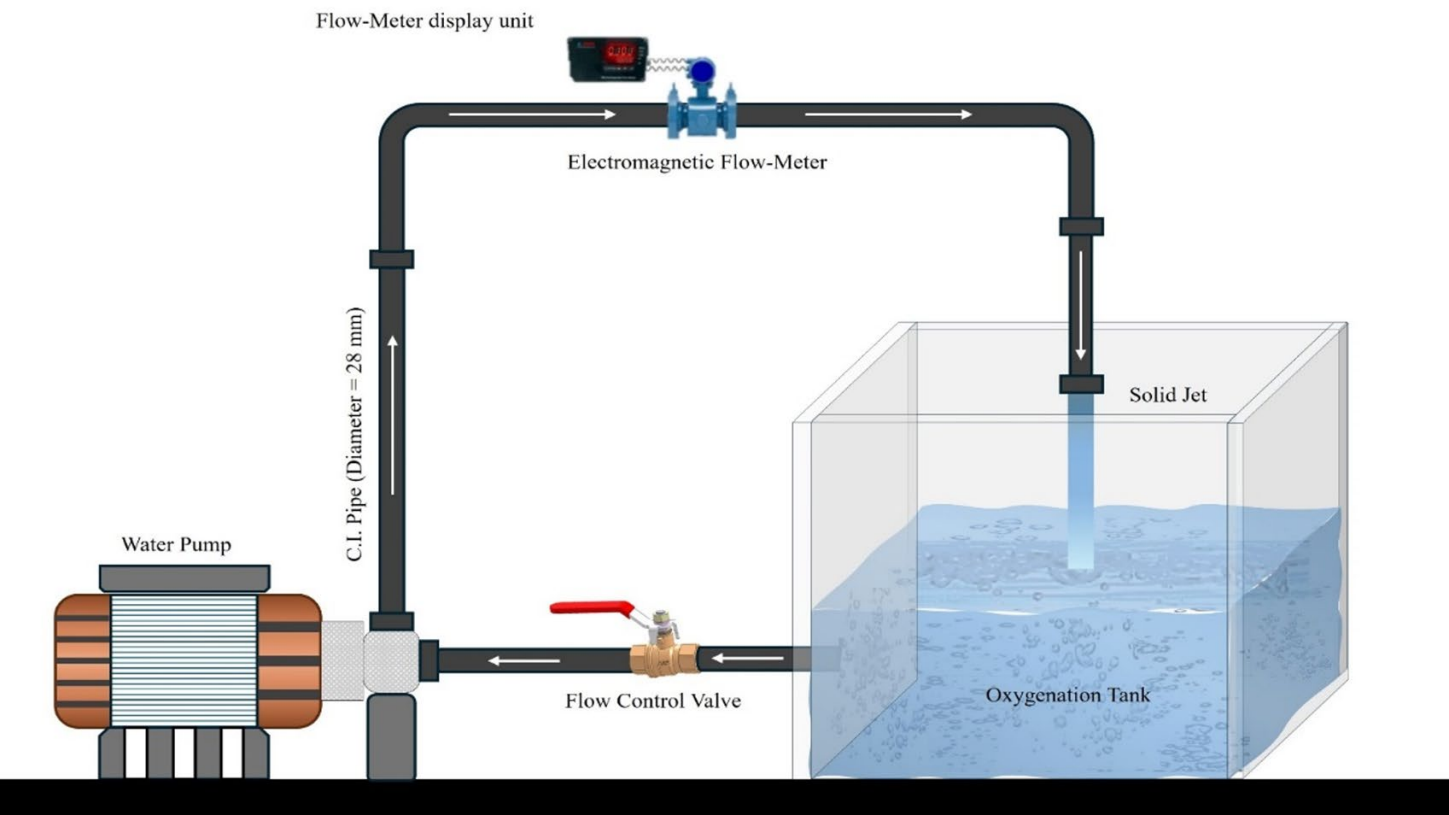
Schematic representation of the experimental arrangement implemented in this study (created using Microsoft PowerPoint for Microsoft 365 MSO, version 2509; available at https://www.microsoft.com/en-us/microsoft-365/download-office?msockid=1edad389b19960220682c07eb0316166#download).

The experimental arrangement included a transparent water tank made of acrylic sheets, which measured 0.5 m x 0.5 m x 0.6 m in dimension, as shown in Fig. 1. The tank was filled with water using a cast iron (C.I.) pipe measuring 28 mm internal diameter, which was connected to a centrifugal pump rated at 1 horsepower was securely mounted on a robust platform for stability during operation. A valve was installed that controlled the water flow just after the pump’s exit, and an electromagnetic flow measurement device attached to the conduit was used as a gauge for measuring the amount of water being discharged. An additional valve was installed during the experimental procedure to assist in draining the tank, which used tap water. The configuration involved a power source to commence and cease the pump and flowmeter functions. The pump was operated under five distinct flow rate conditions: 3.04 l/s, 2.46 l/s, 1.93 l/s, 0.54 l/s and 1.05 l/s.

### Aeration device

**Fig. 2:**
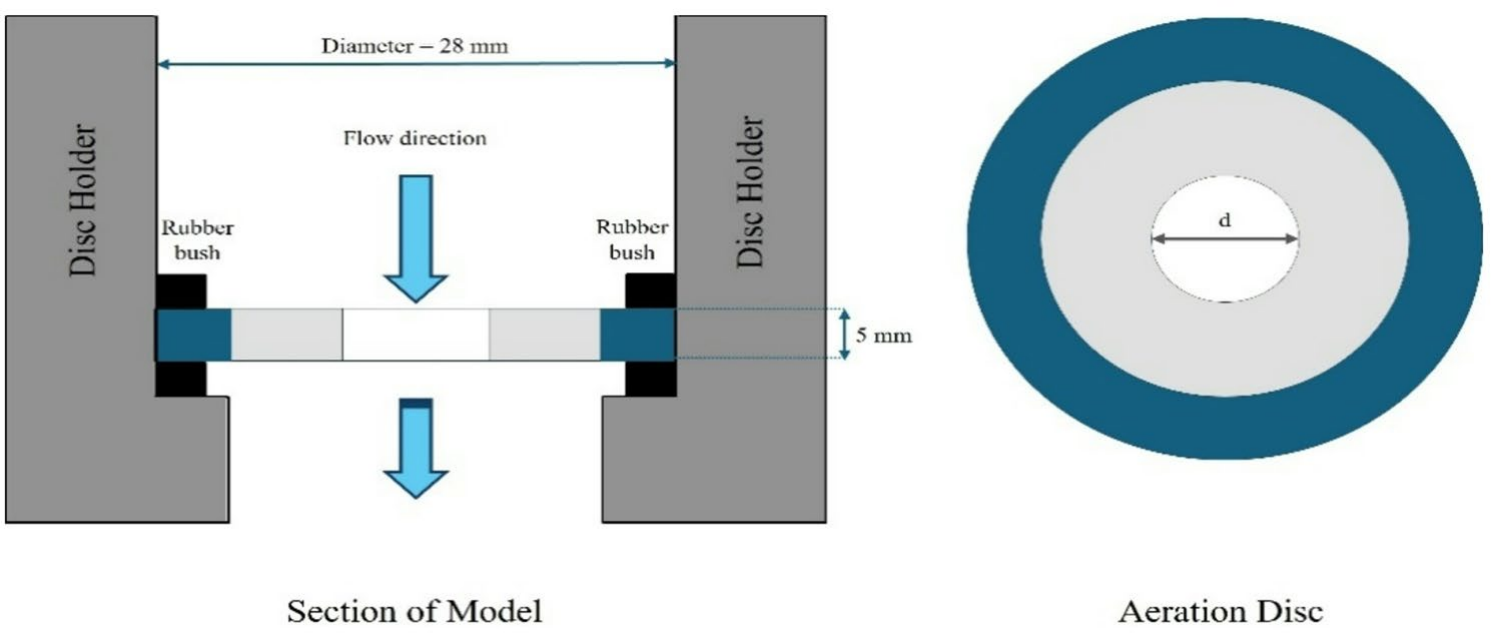
Diagram illustrating the structure of the solid plunging circular jet device (created using Microsoft PowerPoint for Microsoft 365 MSO, version 2509; available at https://www.microsoft.com/en-us/microsoft-365/download-office?msockid=1edad389b19960220682c07eb0316166#download).

The aeration device featured a cylindrical cast iron holder with customized discs, each containing a specific number of openings. The apertures were sealed with rubber gaskets to ensure an airtight connection and affixed to the outlet of the cast iron pipe. The circular jet design was carefully developed, considering the diameter of the jets to define flow area and surface area per unit jet length. The selected diameter influenced flow velocity, aeration efficiency, and system effectiveness. This study explored the effects of varying the number of openings, jet lengths, and intercepted air between jets on oxygen transfer parameters. The aerator discs used in the study were configured with 8, 4, 2 and 1 circular openings, and experiments were conducted using jet lengths of 470 mm, 370 mm, 270 mm and 170 mm. Fig. 2 provides a schematic representation of the circular solid jet device used for the experiments.

Aeration discs were created with forms as specified in Table 1. The area of the openings in the discs was roughly 20%, 16%, 12% and 8% of the cross-sectional area of the pipe. The measurements were rounded to one decimal place to accommodate casting constraints. Table 1 presents the specific characteristics and arrangements of solid jet plunging devices.

**Table 1:** Configuration details of aerator assemblies: circular opening attributes and geometrical properties.

Proportional % of Flow Area Compared to Pipe Area	Flow Area of Jet, (A_f_) (mm^2^)	Diameter of Opening (d) (mm)	Count of Openings per Aerator Disc (*n*)	Flow Discharge (Q) (l/s)	Length of Jet (l) (mm)
8%	49.24	7.92	1	1.05, 1.54, 1.93, 2.46, 3.04	170, 270, 370, 470
		5.60	2		
		3.96	4		
		2.80	8		
12%	73.86	9.70	1		
		6.85	2		
		4.85	4		
		3.43	8		
16%	98.47	11.20	1		
		7.92	2		
		5.60	4		
		3.96	8		
20%	124.03	12.57	1		
		8.90	2		
		6.28	4		
		4.45	8		

**Fig. 3:**
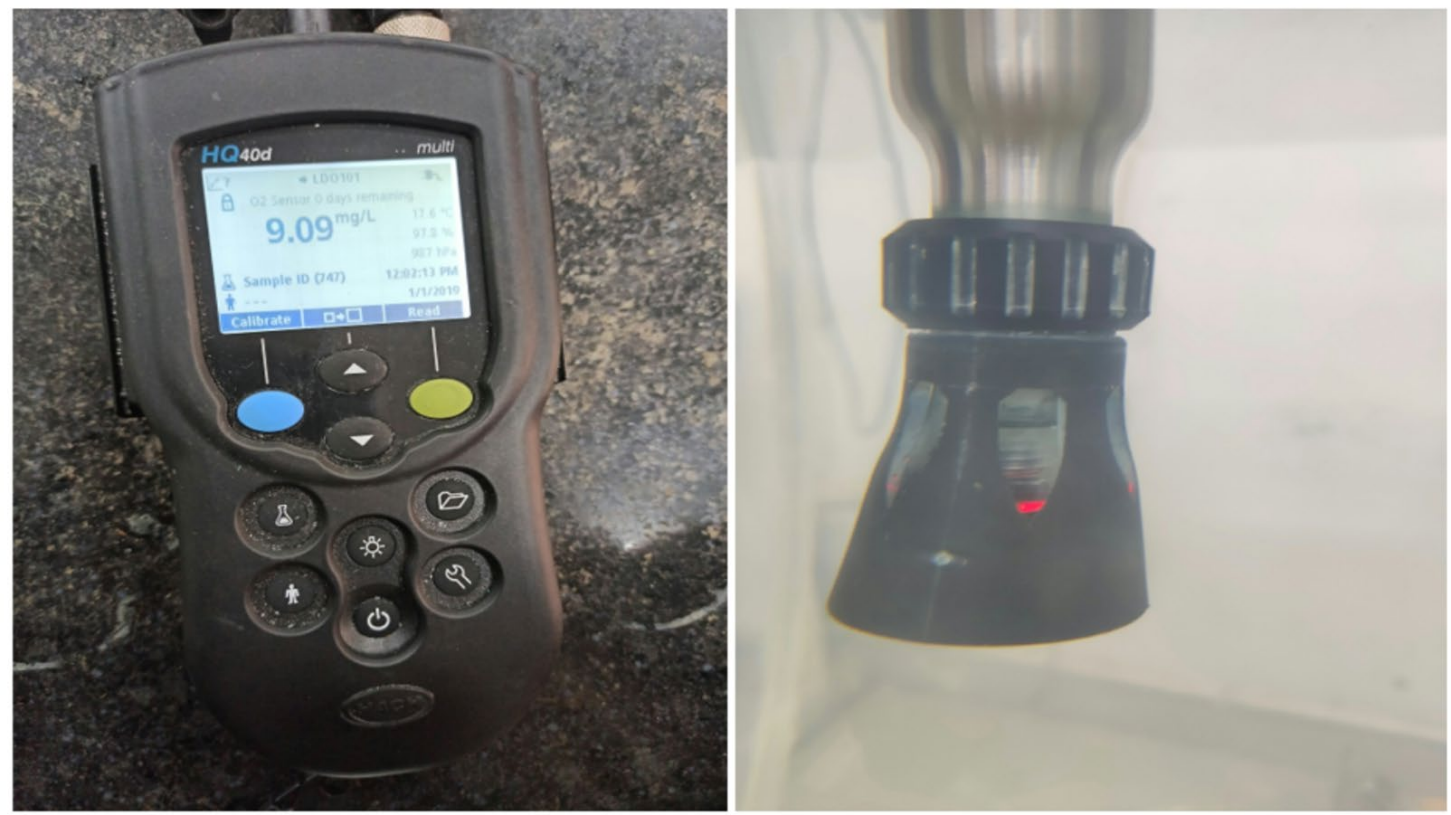
Multimeter (HACH) equipped with the IntelliCAL LDO101 probe.

IntelliCAL LDO101 Optical DO Probe and HACH HQ40D Multimeter (Fig. 3) were essential in measuring the levels of oxygenation in the previously mentioned experimental setup. The LDO sensor was developed to measure dissolved oxygen, which makes it crucial for accurately measuring the effects of aeration^33^. The IntelliCAL LDO101 is distinguished for its precise measurement of dissolved oxygen concentrations, due to its advanced luminous technology. The device’s stirring mechanism is unique and continuously pumps water around the sensor. The presence of this attribute prevents the accumulation of stagnant water, which could lead to incorrect readings of dissolved oxygen. This feature prevents the buildup of stagnant water, which could lead to inaccurate interpretation of dissolved oxygen levels.

The sensor had dual functions applications in the research. Before the aeration process, baseline measurements of dissolved oxygen (DO) were taken. Following the completion of an aeration, the sensor measured the remaining dissolved oxygen (DO) levels, providing essentially unbiased information on the achieved transfer. Also, the IntelliCAL LDO101 had an integrated pressure sensor that ensured accuracy despite air variations. It’s worth noting that the LDO101 sensor provided measurement of temperature as well. The impact of temperature differences on aeration is significant, as the solubility of dissolved oxygen (DO) can be influenced by water temperature. The HACH HQ40D multimeter paired with the IntelliCAL LDO101 optical DO sensor were the primary components of the experimental setup. Its efficacy was clearly demonstrated by the correct determination of initial and subsequent concentrations of dissolved oxygen (DO).

### Data measurement and analysis

Non-steady state methodologies were employed to investigate OTE as a significant performance factor. To achieve accurate measurements of oxygenation, sodium sulphite was utilized to eliminate excess D.O. while cobalt chloride provided the catalyst for precise measurements. The duration of the aeration was adjusted to match the discharge rate, with the aim of providing sufficient oxygen intake within one to two minutes. Rates of transfer of oxygen were determined by calculating the volumetric coefficient of oxygen-transfer, K_L_a_(20)_, standardized to 20 °C and corrected for variations. The volumetric coefficient of oxygen-transfer, K_L_a_t_, quantifies the rate at which oxygen transitions from air into water. The coefficient is determined by computing the natural logarithm of the proportion between the change in fully oxygenated state and beginning oxygen concentration in water (C_s_-C_o_) and the change in fully oxygenated state and ultimate oxygen concentration in water (C_s_-C_t_) during the course of the experiment, t. In this particular framework, C_o_ denotes the level of dissolved oxygen preceding the pump’s operation, C_t_ signifies its concentration after the pumps’ functioning, and C_s_ descripts solely the state of oxygen saturation at temperature t. The equation is provided as follows:

1 $$\:{K}_{L}{a}_{t}=\:\frac{1}{t}\:\text{ln}\left(\frac{{C}_{S}-{C}_{O}}{{C}_{S}-{C}_{t}}\right)$$

To ensure consistency across different aerator designs, $\:{K}_{L}{a}_{t}$ was adjusted to temperature of 20 °C, serving as standard reference. As illustrated in Eq. 2, the temperature-dependent variability of $\:{K}_{L}{a}_{t}$is described in accordance with Daniil and Gulliver^34^.

2 $$\:{K}_{L}{a}_{\left(20\right)}=\:{K}_{L}{a}_{T}\:\times\:{\theta\:}^{(20-T)}$$

Where $\:\theta\:\approx\:1.024\:$for temperature range 5-24^o^ C.

$\:\theta\:\:\approx\:1.028\:$for temperature range 25-34^o^ C.

$\:\theta\:\:\approx\:1.031\:$ for temperature range 35-45^o^ C.

and the coefficient of oxygen-transfer under standard conditions is represented by the symbol $\:{K}_{L}{a}_{\left(20\right)}$, while the coefficient at a specific temperature T (°C) is represented by $\:{K}_{L}{a}_{T}$. The temperature of the water in degrees Celsius is denoted by the parameter T. Equation 3 illustrates the calculation of the energy associated with the flow, which is expressed as jet power (kW/m³)^9^.

3 $$\:\frac{P}{V}=\:\frac{\frac{1}{2}\rho\:Q{{v}_{j}}^{2}}{V}$$

In the equation, the density of the liquid, denoted by ρ, is measured in kg/m³. The jet velocity (*v*_*j*_​​) exiting the nozzle is expressed in m/s, while the discharge (Q) represents the flow rate in m³/s. Equation 4 is used to determine the oxygen transfer rate.

4 $$\:{O}_{R}=\:{K}_{L}{a}_{\left(20\right)}\times\:3600\times\:{{C}_{S}}^{*}$$

Where $\:{{C}_{S}}^{*}\:$ symbolizes the level of oxygen saturation at 20^o^ C. Equation 5 provides a definition for the OTE of an aerator, which is given in kgO_2_/kWh^9^.

5 $$\:O.T.E.=\:\frac{{O}_{R}\times\:V}{P}$$

### Overview of computational intelligence techniques

It is important to be able to use machine learning (ML) and artificial intelligence (AI) methods without having to do a lot of programming in order to create models and algorithms that can find patterns and make smart choices. The sections that follow give short descriptions of the machine learning methods that were used in this work. These algorithms were deliberately selected to span baseline linear regression, interpretable regression trees, and advanced ensemble methods, thereby enabling both benchmarking and robust predictive modelling of OTE dynamics.

#### Review on M5P tree technique

Model trees are in essence regression trees that utilise linear functions at their end nodes^23^. These trees function in a manner that is analogous to piece-wise linear equations. Quinlan^19^ defines the M5P model tree as a decision tree that branches into two halves and uses linear equations at its endpoints to estimate unremitting mathematical properties. In order to lessen the prospect of overfitting, the process of pruning is integrated into the design. Every node utilises a separation technique to improve the precision of the subsets by reducing variations within each branch. An M5P model is constructed through three essential stages: tree development, pruning, and smoothing. To create the model tree, one must estimate the standard deviation values at the nodes. This is done by applying specified separation criteria. This method generates linear functions at each node, and the standard deviation methodology is used to calculate the anticipated error at the terminal node.

Equation 6 provides standard reduction.

6 $$\:\text{S}\text{D}\text{R}={s}_{d}\left(\text{Z}\right)-{\sum\:}_{\text{i}}^{\text{n}}\left(\frac{|{\text{Z}}_{\text{i}|}}{\left|\text{Z}\right|}{s}_{d}({Z}_{i}\right)$$

Where Z represents a collection of examples at the Z_i_ node that demonstrate the result of a subset of feasible set examples. S_d_ refers to the standard equation. This method produces a remarkable tree structure that has a high level of accuracy in making predictions.

#### Review on random forest technique (RF)

Breiman^29^ pioneered the development of the Random Forest method, a versatile technique that is extensively used to solve a variety of nonlinear and complicated engineering problems. This method entails the creation of many decision trees, each derived from a distinct bootstrap (bagging) sample of the original dataset. The method divides each node based on a random subset of the given parameters. The Random Forest method is easy to apply and operates well independent of the training set’s features, resulting in excellent prediction accuracy^21,35^. It involves the definition of two critical parameters: the number of input parameters (m) and the number of trees (k). Model development is often done using a trial-and-error technique. WEKA 3.9 software was used to implement the Random Forest model in this study.

#### Reduced error pruning (REP) tree technique: review

The REP Tree Classifier utilises entropy-based data gathering and variance error minimization methods to generate decision trees efficiently^23^. This classifier iteratively generates several updates to regression trees and then selects the most effective tree from the generated iterations. The algorithm constructs a regression/decision tree by employing variance and information gain. By establishing connections between various forms and utilising pruning calculations that rely on the anticipated inaccuracy of the tree in a typical situation, it effectively decreases the rate of pruning errors. During the first modelling process, the numerical attributes are organised in a sorted manner. The method subsequently divides the samples and manages any absent values^36^.

#### Review on random tree (RT)

The Random Tree algorithm, devoid of pruning, assesses a predetermined quantity of random attributes at every node. Random Tree (RT) algorithms primarily depend on arbitrary selection and have limited associations with traditional machine learning principles^37^. The random forest algorithm utilises the technique of bagging, where each node is partitioned among randomly selected predecessor subsets in an efficient manner. The algorithm efficiently manages both classification and regression tasks. Random Trees consist of a collection of tree estimators. During the process of classification, the Random Tree classifier evaluates the input vector for each tree in the forest and identifies the category that receives the highest number of votes. In the event of a tie, the classifier’s answer is determined by calculating the average output of all the trees in the forest^38^. RTs are a combination of two machine learning algorithms: Random Forest (RF) and single model trees. Model trees consist of decision trees that have linear models at each leaf node, which are specifically designed to match the local subdomain represented by that particular leaf. Research has demonstrated that incorporating individual stable trees can greatly improve the effectiveness of Random Forests. The introduction of diversity among trees is achieved by two randomization techniques: firstly, by sampling the training data for each tree using bagging, and secondly, by evaluating only a random subset of characteristics for each node during the tree growth process, and selecting the best split from that subset. Random Model Trees combine the principles of Random Forests and model trees for the first time. Random Trees (RTs) employ this method to divide criteria, fostering trees that are evenly distributed with a consistent ridge environment throughout all branches. This simplifies the process of optimisation^39^.

#### Linear regression (LR) technique

Linear Regression (LR) is a statistical method employed to ascertain the relationships between a dependent and a number of independent variables. The output variable, represented as Z, is forecasted using the input variables X_1_, X_2_, X_3,_,., X_n_^40^. The equation typically used for linear regression is as follows:

7 $$\:Z=\:{C}_{o}+{C}_{1}{X}_{1}+{C}_{2}{X}_{2}+\dots\:\dots\:\dots\:\dots\:\dots\:\dots\:.+{C}_{n}{X}_{n}$$

## Data set

**Fig. 4:**
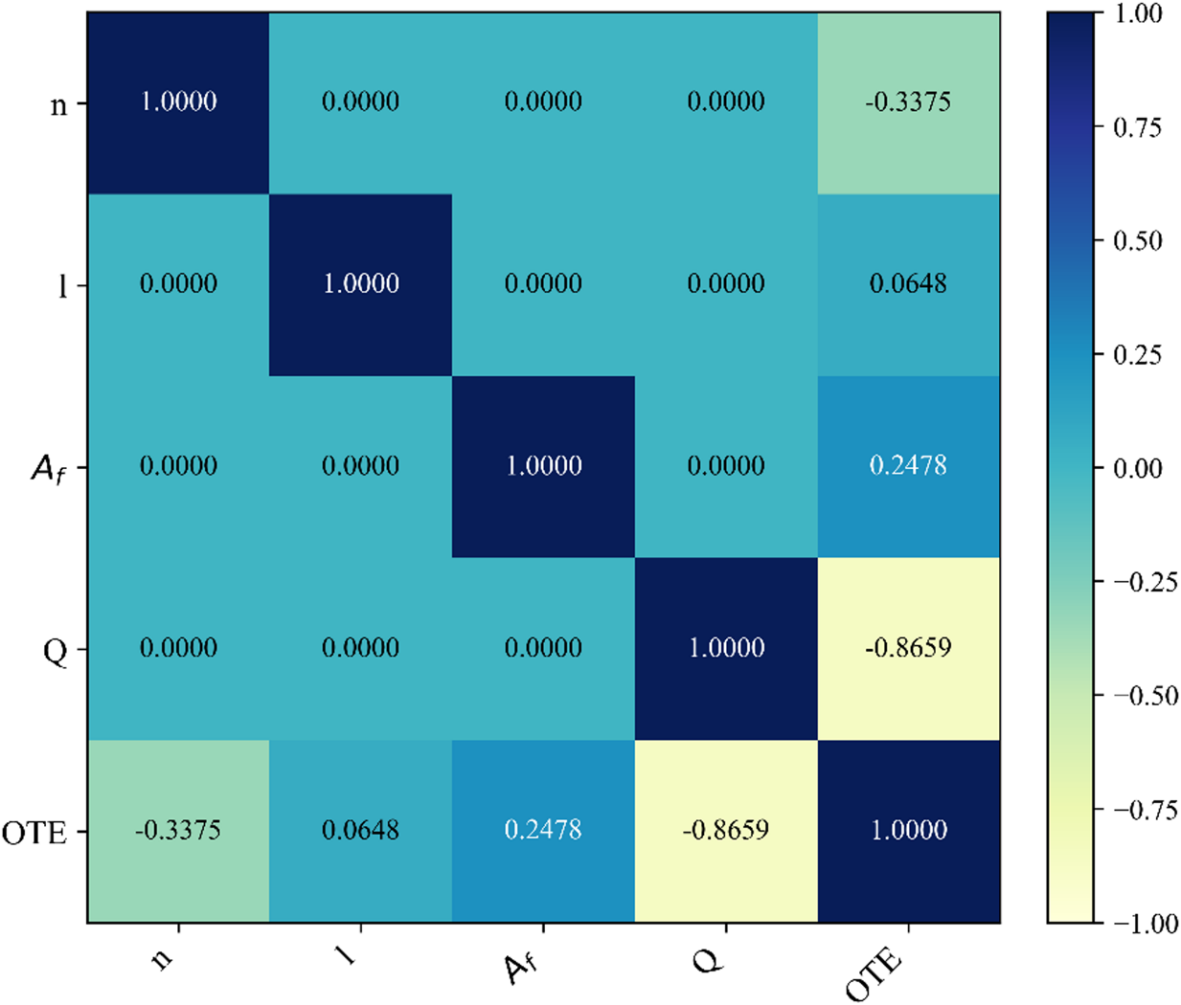
Spearman correlation heatmap of input variables and OTE.

This study focused on predicting the oxygen-transfer efficiency (OTE) of solid-jet aerators with circular apertures using 320 experimental observations reported in Shukla et al.^10^. Predictors were the number of openings, disc flow area, jet length, and discharge rate, with OTE as the response. No normalization was applied and the dataset contained no missing values; records were randomly shuffled. The dataset was partitioned 70/30 (224/96) into training/testing using a fixed random seed (42). Models—LR, M5P, RF, REP-Tree, and RT—were trained on the 224-sample training set, with 96 held out for testing and validation. Fig. 4 presents the Spearman rank correlation heatmap illustrating the monotonic relationships between the input parameters and the target output (OTE). The analysis indicates that discharge exhibits the strongest negative correlation with OTE, followed by the number of apertures, while flow area and jet length display comparatively weaker positive associations. These correlation patterns provide an initial indication of variable influence and offer a statistical basis for understanding the relative predictive significance of each parameter in subsequent model evaluations. Table 2 presents a comprehensive overview of the data features that were utilised for the creation and validation of the model.

**Table 2:** Statistical evaluation of training and testing data set.

Descriptive statistics	Minimum	Maximum	Mean	Standard Deviation	Kurtosis	Skewness	Confidence Level (95.0%)
**Training data set (224)**							
*Aerator openings (n)*	1	8	3.74	2.72	-1.14	0.65	0.36
*Length of jet (l) (mm)*	170	470	324.91	109.52	-1.3	-0.06	14.42
*Flow area [A*_*f*_ *(mm*^*2*^*)]*	49.24	124.03	86.4	27.84	-1.31	-0.03	0.00
*Discharge [Q (l/s)]*	1.05	3.04	2.04	0.69	-1.24	0.11	0.09
*OTE (kgO*_*2*_ */kW-hr)*	0.28	30.95	5.32	6	4.06	2.05	0.79
**Testing data set (96)**							
*Aerator openings (n)*	1	8	3.78	2.61	-0.99	0.7	0.53
*Length of jet (l) (mm)*	170	470	308.54	117.31	-1.46	0.16	23.77
*Flow area [A*_*f*_ *(mm*^*2*^*)]*	49.24	124.03	86.4	27.84	-1.47	0.07	0
*Discharge [Q (l/s)]*	1.05	3.04	1.92	0.7	-1.18	0.24	0.14
*OTE (kgO*_*2*_ */kW-hr)*	0.25	27.45	5.91	6.41	1.51	1.53	1.3

## Models formation

This work utilised five modelling strategies, namely LR, M5P, RF, REP Tree and RT, through the use of WEKA 3.9 software. These techniques were applied based on experimental data.

### Parameters for performance appraisal

The accuracy of the implemented models was evaluated using several statistical measures, such as Root Mean Squared Error (RMSE), Scattering Index (SI), Coefficient of Correlation (CC), Mean Absolute Error (MAE), and Nash-Sutcliffe Model Efficiency (NSE). In order to achieve the best model, it is desirable for the RMSE, SI, and MAE error values to be minimised, while the CC and NSE values should be close to 1. The quantification of these five performance assessment indicators was conducted via Eq. (8) through (12).

8 $$\:CC=\:\frac{N\sum\:_{i=1}^{N}\left({G}_{i}{P}_{i}\right)-(\sum\:_{i=1}^{N}{G}_{i}\left)\right(\sum\:_{i=1}^{N}{P}_{i})}{\sqrt{N(\sum\:_{i=1}^{N}{{G}_{i}}^{2})-{(\sum\:_{i=1}^{N}{G}_{i})}^{2}}\sqrt{N(\sum\:_{i=1}^{N}{{P}_{i}}^{2})-{(\sum\:_{i=1}^{N}{P}_{i})}^{2}}}$$

9 $$\:MAE=\frac{1}{N}\sum\:_{i=1}^{N}\left|{P}_{i}-{G}_{i}\right|$$

10 $$\:RMSE=\sqrt{\frac{1}{N}(\sum\:_{i=1}^{N}{\left({P}_{i}-{G}_{i}\right)}^{2}}$$

11 $$\:SI\:=\:\frac{\sqrt{\frac{1}{N}{\sum\:}_{i=0}^{N}({{{P}_{i}-G}_{i})}^{2}}}{\stackrel{-}{{G}_{i}}}$$

12 $$\:NSE\:=1-\lceil\:\:\frac{{\sum\:}_{i=1}^{N}{({P}_{i}-G)}^{2}}{{\sum\:}_{i=1}^{N}{({P}_{i}-{G}_{i})}^{2}}\rceil\:$$

Where N is the number of data sets, $\:{G}_{i}$ represents the actual values, and $\:{P}_{i}$ represents the estimated values, whereas $\:\stackrel{-}{{G}_{i}}$ denotes average of actual values.

### Modelling workflow and reproducibility checklist

#### Workflow

**Fig. 5:**
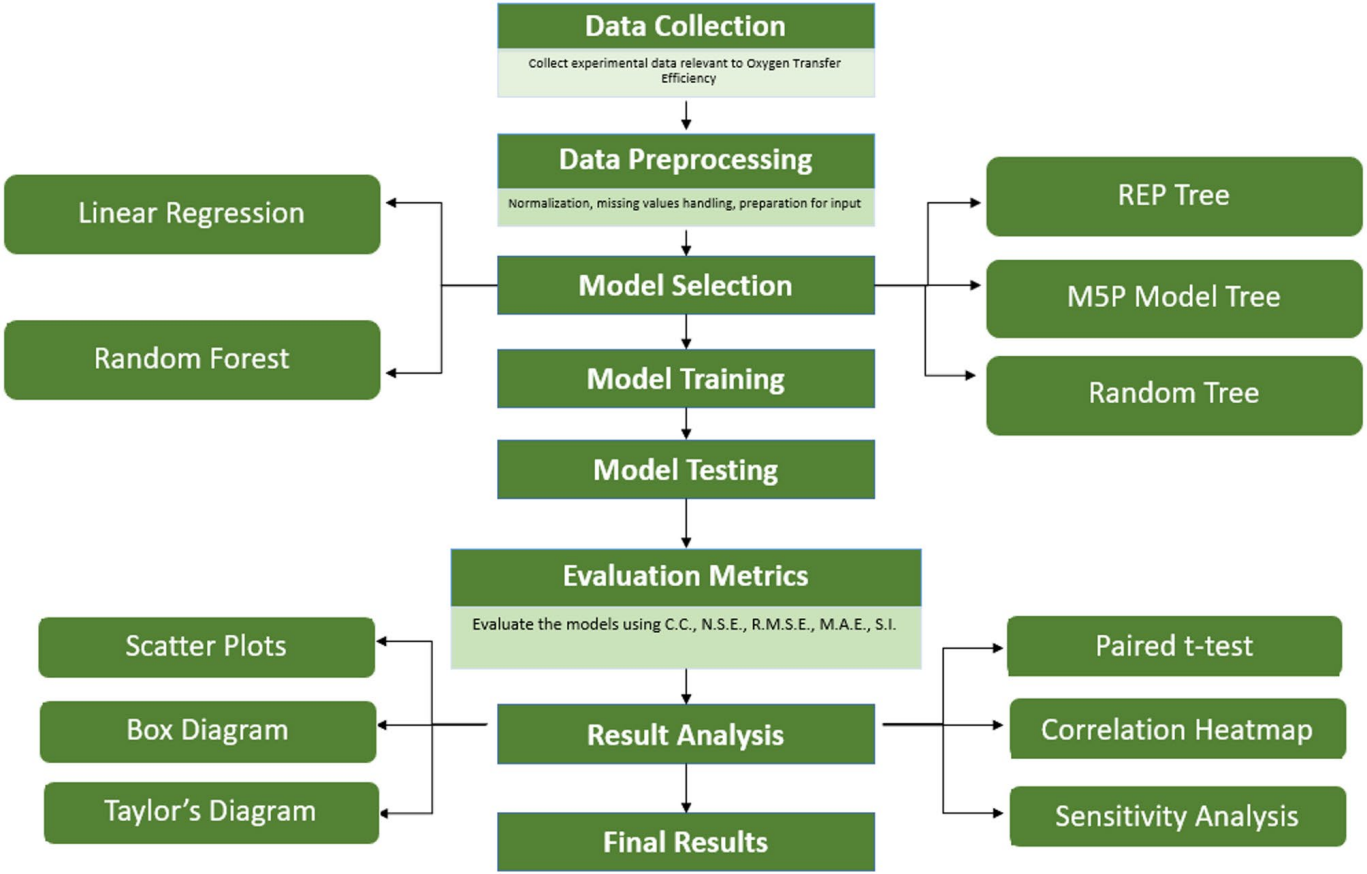
Methodological workflow for modelling oxygen-transfer efficiency (OTE) of circular solid-jet aerators.

Fig 5 summarizes the end-to-end procedure used in this study. Experimental OTE data were compiled for circular solid-jet aerators and then preprocessed (normalization, handling of missing values, formatting of inputs). Five algorithms were considered: Linear Regression (LR), M5P model tree, Random Tree (RT), Reduced Error Pruning (REP) Tree, and Random Forest (RF). Models were trained on the calibration subset and assessed on the held-out test subset. Performance was quantified using the coefficient of correlation (CC), Nash–Sutcliffe efficiency (NSE), root mean squared error (RMSE), mean absolute error (MAE) and scattering index (SI). Result analysis comprised agreement scatter plots, box plots, a Taylor diagram, a paired t-test to compare model errors, and a Spearman correlation heatmap to examine concordance among model predictions and observations. Sensitivity analysis was performed to identify the most influential inputs. The final model was selected based on test-set performance and generalization.

#### Reproducibility checklist

To ensure methodological transparency and experimental reproducibility, a detailed reproducibility checklist has been included in Table 3. This checklist systematically outlines the dataset structure, preprocessing pipeline, model configuration, training procedure, evaluation metrics, and visualization protocols adopted in this study. Each item specifies where the corresponding details are described in the manuscript, thereby enabling independent validation and replication of the presented results. The study maintained consistent computational environments, fixed random seeds, and standardized protocols for model training and testing to minimize variability in outcomes. Moreover, all figure generation procedures were documented using identical datasets and consistent visualization tools to maintain statistical fidelity.

**Table 3:** Reproducibility checklist for the developed machine learning framework applied to OTE prediction.

Item	Description	Location in Manuscript
Data Source and Composition	Experimental dataset consisting of 320 observations obtained from circular solid-jet aerators under varying discharge (1.05–3.04 l/s), jet length (170–470 mm), number of apertures (1–8), and opening area (49.24–124.03 mm²).	Section Experimental configuration and setup–Aeration device
Input and Output Variables	Inputs: jet discharge (Q), number of openings (n), jet length (l), opening area (A_f_). Output: measured oxygen transfer efficiency (OTE).	Section Experimental configuration and setup–Data measurement and analysis
Data Preprocessing	No normalization; no missing values; randomly shuffled before before training.	Section Data set
Data Partitioning	Dataset divided into 70% training and 30% testing subsets using random seed = 42 for reproducibility.	Section Data set
Software and Environment	Modelling conducted in WEKA 3.9 under Windows 11 (64-bit), Intel i7 (3.0 GHz), 32 GB RAM. Statistical plots generated in OriginPro 2024; flowchart prepared in PowerPoint 2021; heatmap prepared through python.	Section Models formation
Machine Learning Algorithms	Five regression algorithms used: Linear Regression (LR), M5P Model Tree, Random Tree (RT), Reduced Error Pruning (REP) Tree, and Random Forest (RF).	Section Overview of Computational Intelligence Techniques
Model Hyperparameters	LR: default settings; M5P: m = 4, pruning = True; RT: Kvalue = 10; REP Tree: default settings; RF: m = 1, K = 10.	Section Assessment of the LR based model–Assessment of the RT based model
Training Protocol	Each model trained on 70% subset; 10-fold cross-validation performed during tuning. Mean and standard deviation of all metrics computed across folds.	Section Data set
Evaluation Metrics	Coefficient of Correlation (CC), Mean Absolute Error (MAE), Root Mean Square Error (RMSE), Scattering Index (SI), and Nash–Sutcliffe Efficiency (NSE).	Section Parameters for Performance Appraisal
Statistical Significance Testing	Paired t-test performed on model residuals (testing dataset) at α = 0.05 to determine performance differences; correlation heatmap computed using Spearman’s ρ.	Section Inter comparison amongst applied models
Result Visualization	Heatmap prepared through python programming; Scatter plots prepared in Excel; box and Taylor diagrams generated in OriginPro 2024 for consistent statistical representation; flowchart illustrated in PowerPoint 2021.	Sections Data set, Modelling workflow and reproducibility checklist, Assessment of the LR based model–Inter comparison amongst applied models
Sensitivity Analysis	Model sensitivity assessed using a leave-one-input-out (LOIO) approach in WEKA, maintaining identical hyperparameters for each iteration. Relative decrease in predictive accuracy after omitting each input variable was used to rank their influence on OTE prediction.	Section Sensitivity analysis
Randomization and Reproducibility	All random operations executed using fixed seed = 42; same seed applied to model initialization and train/test splitting.	Section Data set
Assumptions and Limitations	Experiments conducted under variable ambient temperatures and steady hydraulic head, all oxygen transfer results standardized to 20 °C using the temperature correction equation; results best apply within tested flow regimes.	Section Experimental configuration and setup–Data measurement and analysis
Code Availability	WEKA configuration files and OriginPro plot templates archived and available on request.	Available upon reasonable request from the corresponding author
Hardware and Run Time	Total computation time ≈ 0.9 s (LR) to 4.8 s (RF) on specified hardware; negligible memory overhead.	Available upon reasonable request from the corresponding author
Data and Figure Reproducibility	Scatter plots prepared in Excel; box and Taylor diagrams reproduced in OriginPro using identical data files and scales; flowchart replicated from PowerPoint source using consistent layout and font settings.	Available upon reasonable request from the corresponding author

## Results and discussion

### Assessment of the LR based model

This study focused on developing linear-regression models to forecast the oxygen-transfer efficiency (OTE) of solid-jet aerators with circular apertures. Model development was implemented in WEKA 3.9 using a systematic, cross-validated specification search. The Linear Regression (LR) estimator, grounded in ordinary least squares (OLS), was expressed in the linear form given in Eq. 13.

13 $${\text{OTE}}\,=\, - \,0.{\text{6313 n}}\,+\,0.00{\text{34 l}}\,+\,{\text{48693}}.0{\text{614 }}{{\text{A}}_{\text{f}}} - \,{\text{6}}.{\text{2}}0{\text{24 Q}}\,+\,{\text{15}}.00{\text{16}}$$

**Fig. 6:**
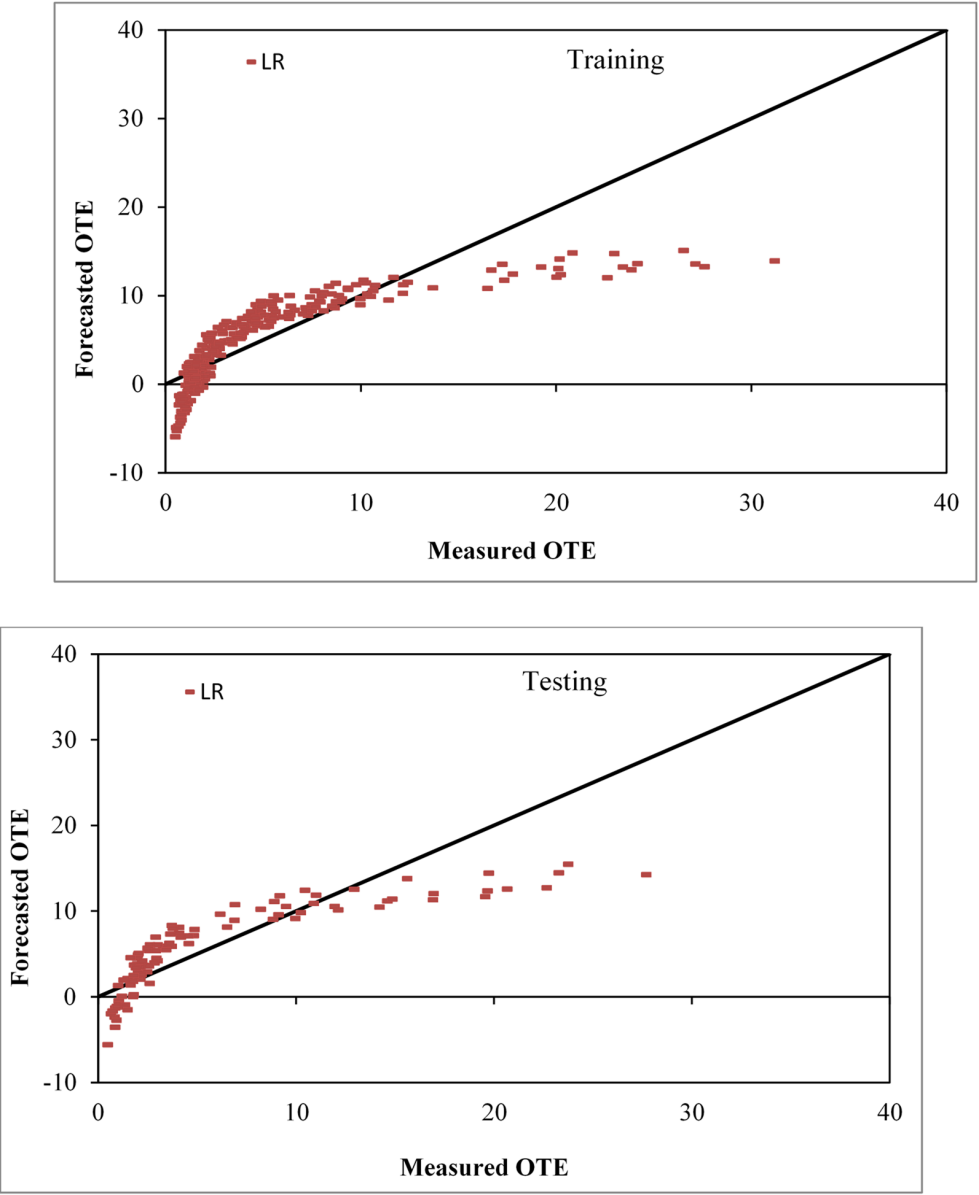
Comparison plot of observed and predicted OTE values for circular jet aerators during training and testing phases correspondingly by the LR based model.

The LR-based models were evaluated for effectiveness using five statistical performance metrics: CC, NSE, RMSE, SI and MAE. The LR-based model demonstrated good performance in both the training and testing stages. The results obtained were as follows: CC = 0.8052 and 0.8376, NSE = 0.6483 and 0.6953, RMSE = 3.5506 and 3.5201, SI = 0.6676 and 0.5956, and MAE = 2.6020 and 2.7532. Fig. 6 displays agreement plots for the training and testing datasets, which compare the real and predicted values of OTE of circular aperture aerators producing solid jet using the LR-based technique. According to the graph, several projected values using the LR-based model were negative throughout both the training and testing phases.

### Assessment of the M5P based model

**Fig. 7:**
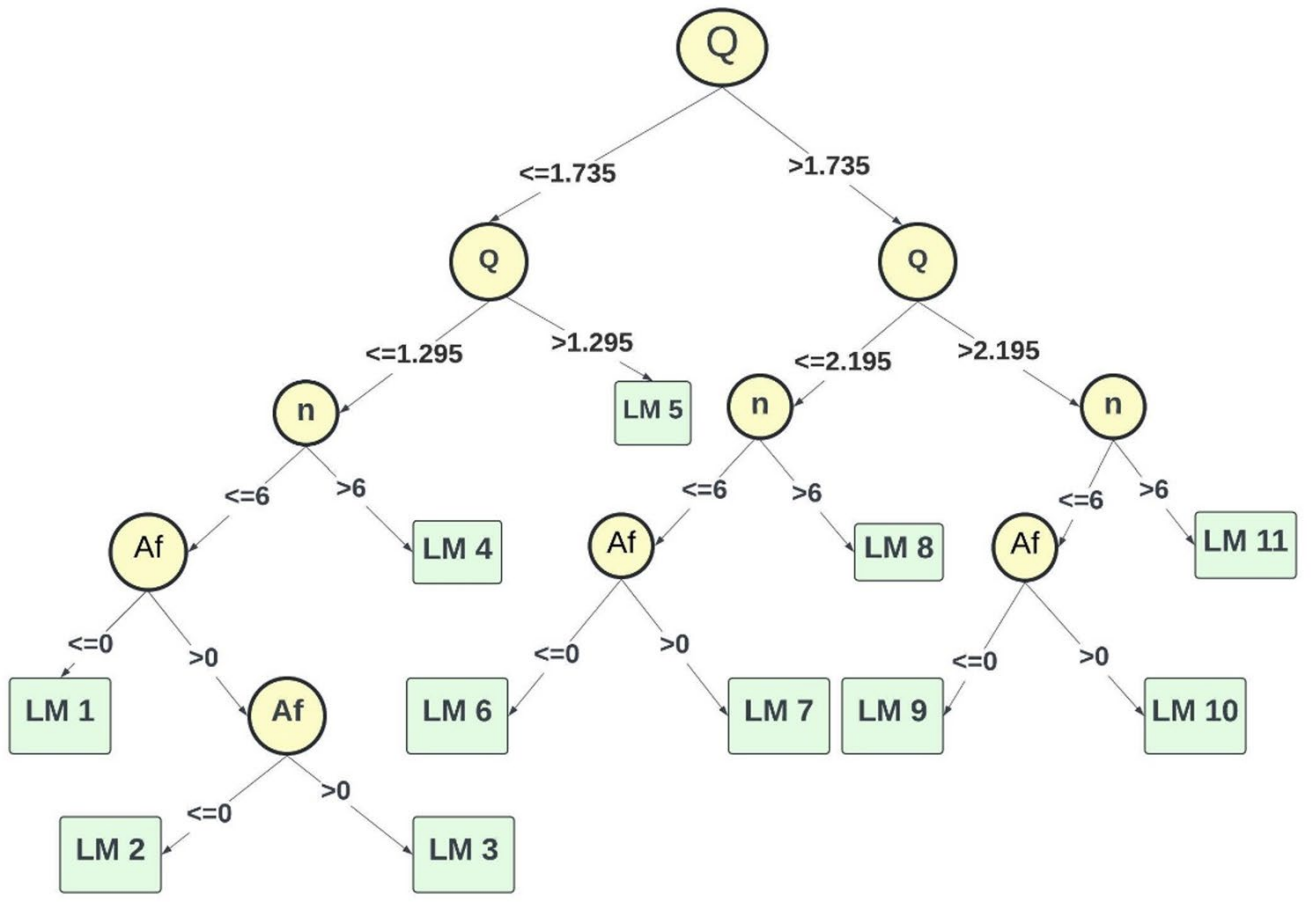
Layout of the M5P model structure.

An M5P model tree was developed via an iterative, cross-validated specification search, with the final setting m = 4 (number of instances) selected on the basis of performance stability and parsimony. A pruned M5P formulation was adopted, and the learned tree structures are shown in Fig. 7.

**Fig. 8:**
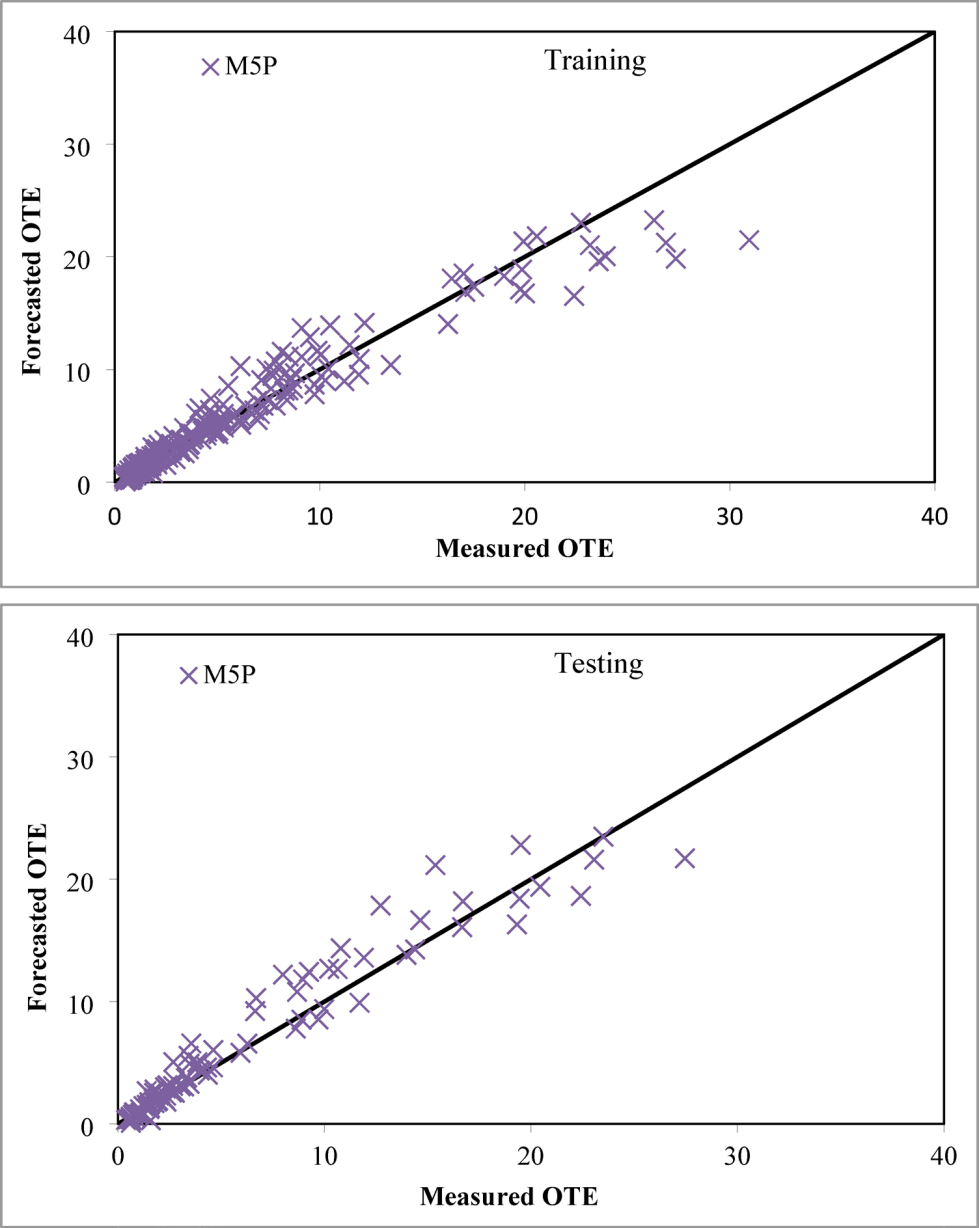
Comparison plot of observed and predicted OTE values for circular jet aerators during training and testing phases correspondingly by the M5P based model.

The linear equations derived from the pruned M5P-based model are presented in Table 4, corresponding to the M5P structure depicted in Fig. 7. Fig. 8 displays graphs that compare the measured and forecasted oxygenation efficiency of solid jet aerators with circular apertures during both the training and testing phases. The projected values obtained from the M5P-based model closely correspond to the line of perfect agreement. The M5P-based model performs better than the LR-based model. It achieves CC values of 0.9685 and 0.9678, NSE values of 0.9351 and 0.9306, RMSE values of 1.5248 and 1.6804, SI values of 0.2867 and 0.2843 and MAE values of 0.8928 and 1.0610 for the training and testing stages, correspondingly.

**Table 4:** Linear equations developed utilising model based on M5P algorithm.

LM number	Linear Equations
1	OTE = -1.4886n + 0.0022 l + 104985.8704A_f_ − 5.0071Q + 14.8793
2	OTE = -1.5339n + 0.0022 l + 88222.735A_f_ − 5.0071Q + 18.0856
3	OTE = -1.6513n + 0.0022 l + 71765.6262A_f_ − 5.0071Q + 20.4973
4	OTE = -1.1151n + 0.0042 l + 88420.8482A_f_ − 5.0071Q + 12.8686
5	OTE = -0.8714n + 0.0051 l + 71281.6908A_f_ − 4.4799Q + 9.2784
6	OTE = -0.3941n + 0.002 l + 41778.9092A_f_ − 1.0867Q + 3.2064
7	OTE = -0.42n + 0.0023 l + 12292.1522A_f_ − 1.0867Q + 5.9539
8	OTE = -0.2834n + 0.0013 l + 25065.2352 A_f_ − 1.0867Q + 3.7343
9	OTE = -0.173n + 0.0008 l + 13110.1531A_f_ − 1.3567Q + 4.1834
10	OTE = -0.2491n + 0.0013 l + 14126.8837A_f_ − 1.9067Q + 5.9808
11	OTE = -0.1455n + 0.0008 l + 10831.4143A_f_ − 1.1723Q + 3.63

The results also reveal that the M5P framework shows a relatively larger negative bias compared with other applied models. This behaviour originates from the pruned regression tree structure of M5P, where localized linear models are fitted after recursive partitioning of the input space. In almost all derived equations (Table 4), the discharge term (Q) carries a consistently negative coefficient of comparatively high magnitude. This systematic weighting leads the model to underestimate OTE values, particularly in scenarios involving higher discharge rates. Although the prediction plots (Fig. 8) confirm that the model aligns well with the line of perfect agreement, this underestimation is evident in the descriptive statistics (Table 6), where the predicted minimum values fall below the observed data. Despite this bias, the model maintains strong predictive ability (CC > 0.96 in both stages) and surpasses the conventional linear regression baseline. However, its limited ability to fully capture nonlinear interactions between discharge, jet length, and aperture geometry explains why ensemble methods such as RF and RT outperform it.

### Assessment of the RF based technique

**Fig. 9:**
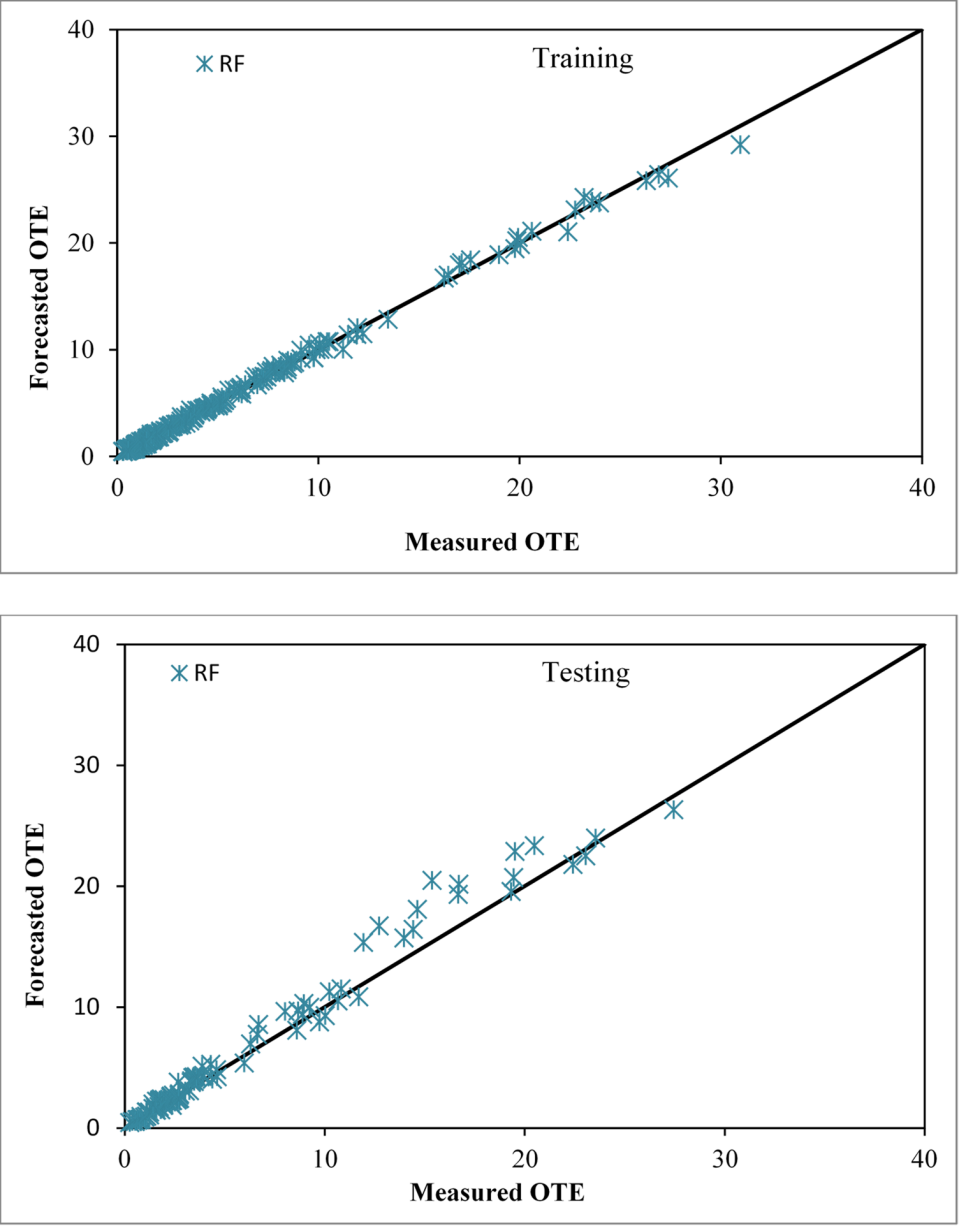
Comparison plot of observed and predicted OTE values for circular jet aerators during training and testing phases correspondingly by the RF based model.

The Random Forest (RF) development followed the same cross-validated specification search as the M5P model, with the optimal configuration obtained at m = 1 (number of features considered at each split) and K = 10 (number of trees in the forest). Fig. 9 displays graphs illustrating the concordance between the real and projected values of oxygenation efficiency for solid jet aerators with circular apertures, throughout both the training and testing phases. The estimated values derived from the RF-based model closely correspond to the observed values. The forecasted values obtained using the RF-based methodology mostly coincide with the line of perfect concordance.

The RF-based method outperforms the M5P and LR-based models, according to the performance evaluation measures. Here are the outcomes of the training and testing phases for the RF-based method: With CC values of 0.9985 and 0.9894, NSE values of 0.9968 and 0.9635, RMSE values of 0.3380 and 1.2187, SI values of 0.0635 and 0.2062, and MAE values of 0.2245 and 0.7276, the scattering index and mean absolute error are as follows.

### Assessment of the REP tree based model

**Fig. 10:**
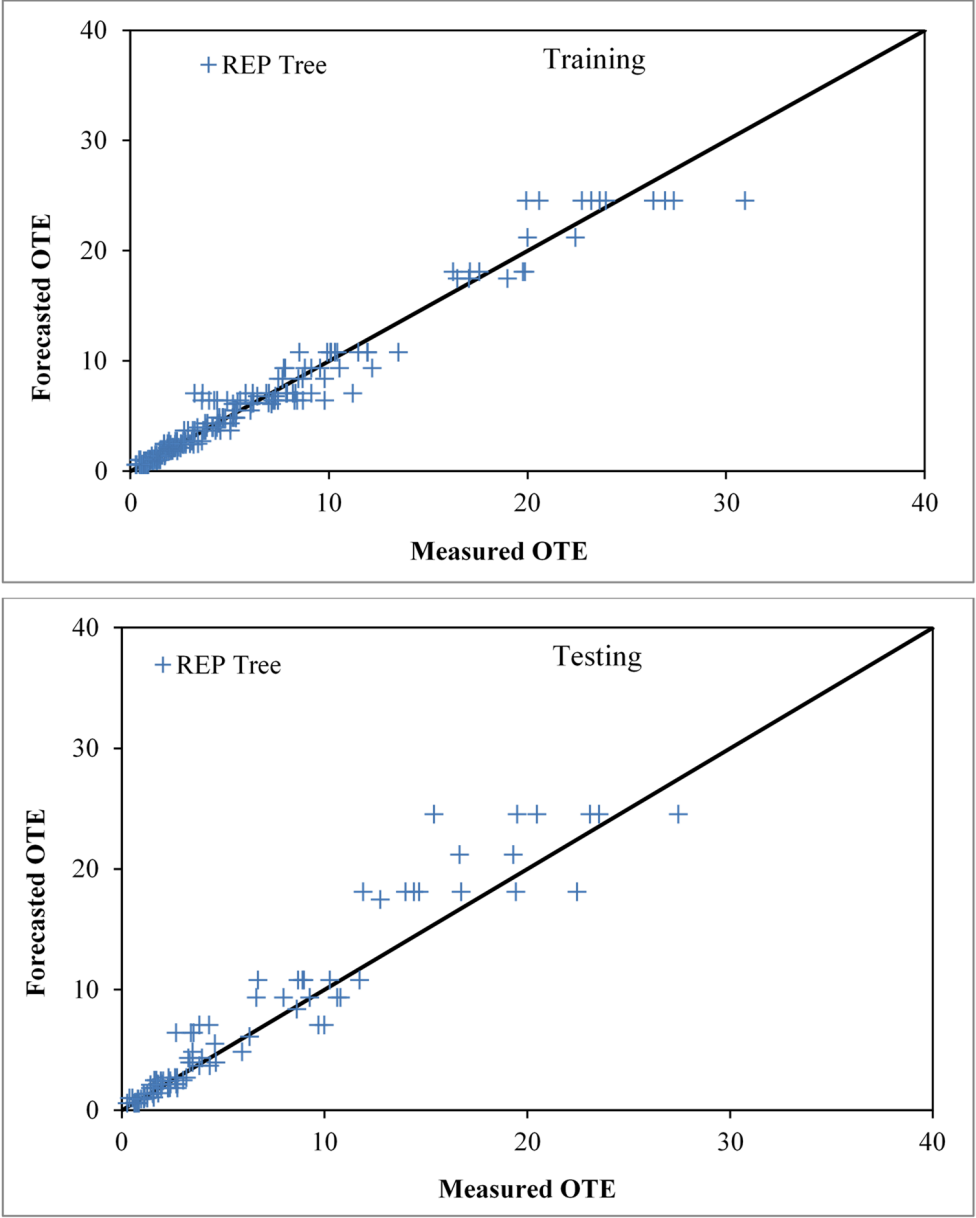
Comparison plot of observed and predicted OTE values for circular jet aerators during training and testing phases correspondingly by the REP tree-based model.

The REP-Tree model for estimating OTE was developed using the same cross-validated workflow as the M5P and RF models in WEKA 3.9, employing the algorithm’s default hyperparameters. The measured and forecasted OTE values are compared in Fig. 10 using the model using the REP tree approach for both training and validation phases. The forecasted values from the REP tree approach align closely with the measured observations. The REP tree approach is effective in predicting OTE, as evidenced by the performance evaluation parameters. The training stage’s CC, NSE, RMSE, SI and MAE values are 0.9823, 0.9649, 1.1219, 0.2110, and 0.6596, respectively. The testing stage’s values are 0.9641, 0.8948, 2.0685, 0.3500, and 1.2748.

### Assessment of the RT based model

**Fig. 11:**
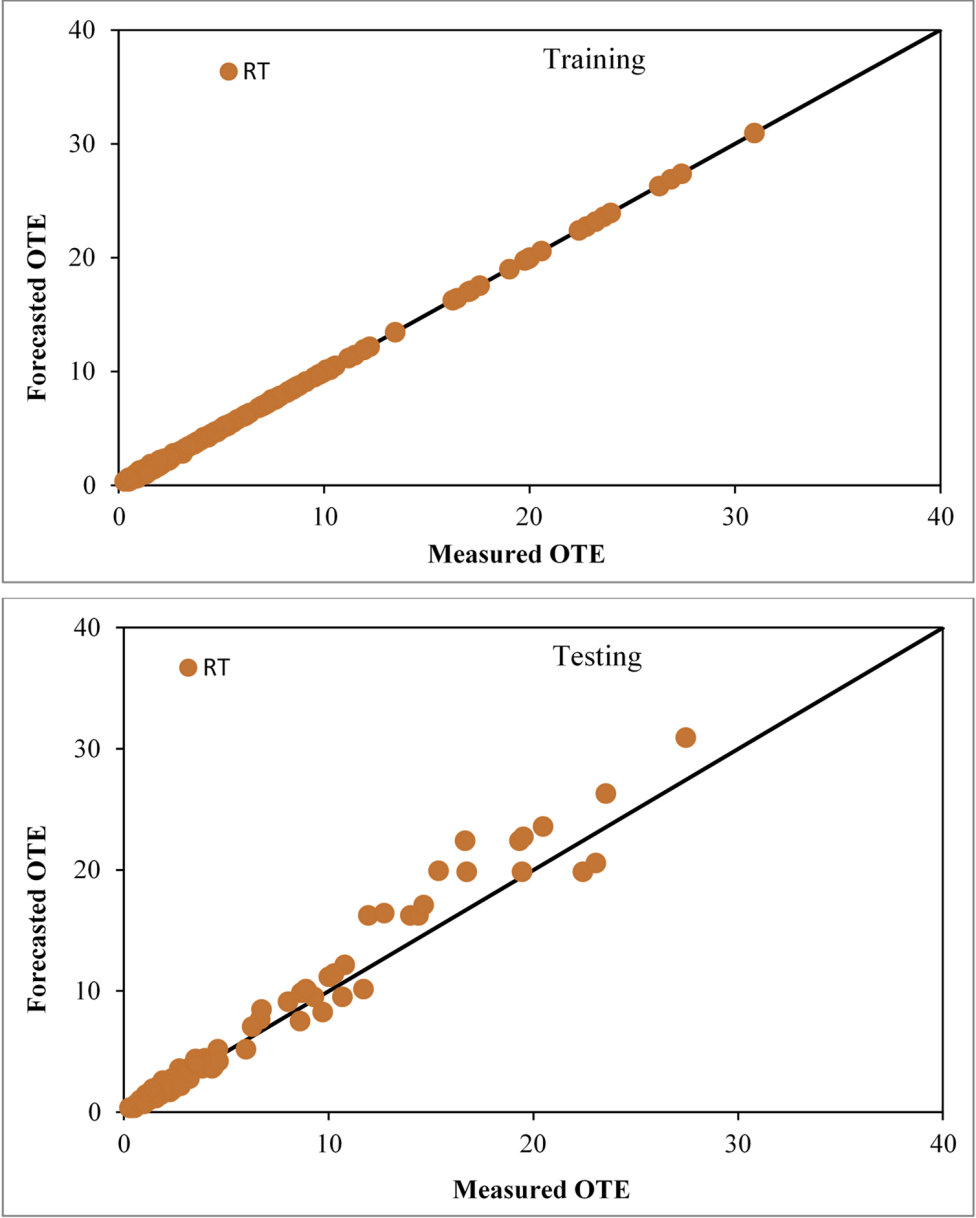
Comparison plot of observed and predicted OTE values for circular jet aerators during training and testing phases correspondingly by the RT based model.

The Random Tree (RT) model was developed via an iterative, cross-validated specification search analogous to the other models, with the final setting KValue = 10 (number of attributes considered at each split) yielding the most reliable predictions. The diagrams in Fig. 11 illustrate the agreement between the actual and predicted OTE of circular openings aerators producing solid jet during both the training and testing sessions. The RT-based technique’s overall performance is suitable for forecasting OTE, as evidenced by CC, NSE, RMSE, SI and MAE values of 0.9999, 0.9997, 0.0978, 0.0184, and 0.0598 for the training stage and 0.9861, 0.9470, 1.4687, 0.2485, and 0.9282 for the testing stage, respectively.

### Inter comparison amongst applied models

**Fig. 12:**
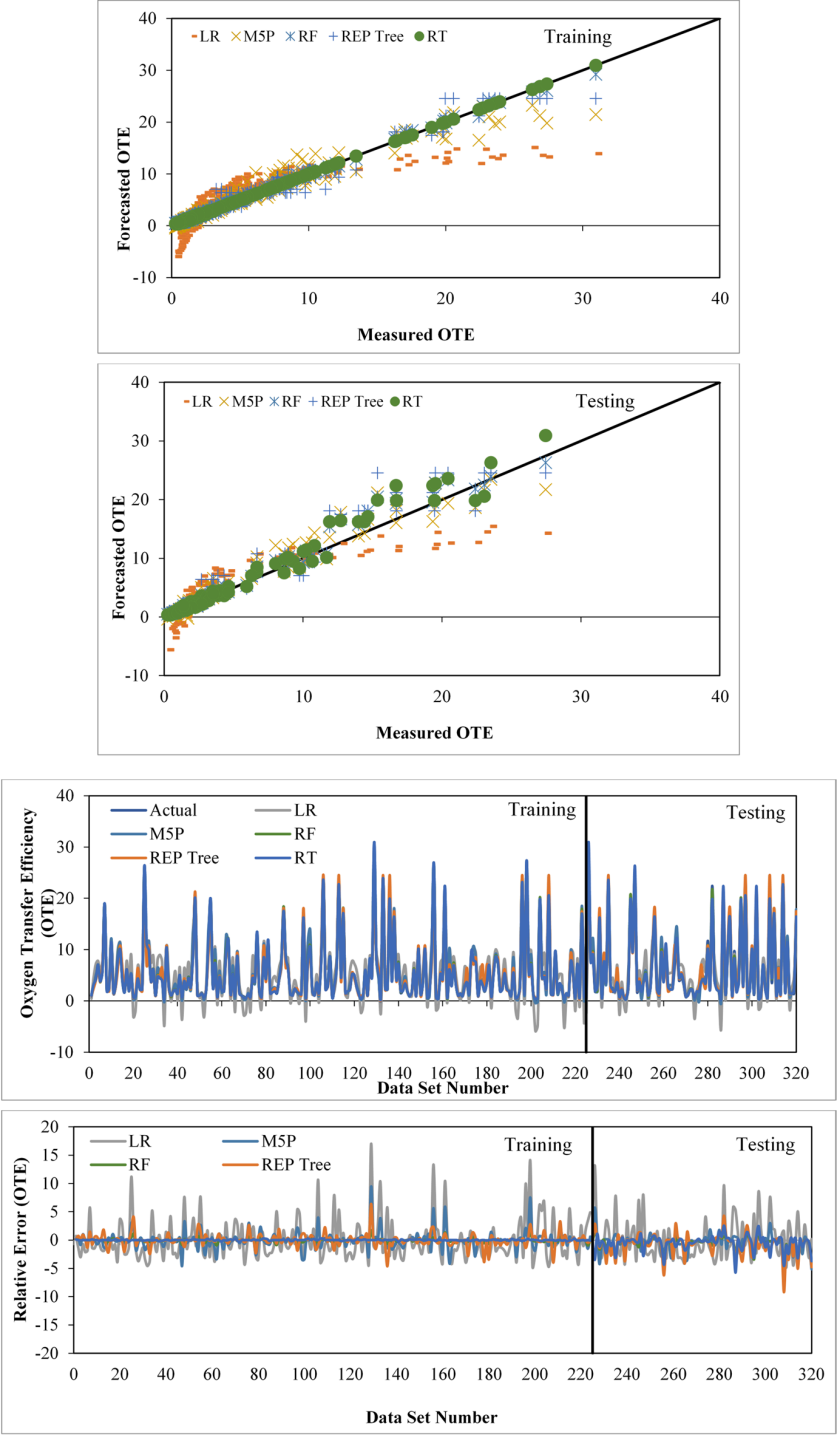
Comparative performance of various computational models for forecasting the OTE of circular jet aerators.

**Fig. 13:**
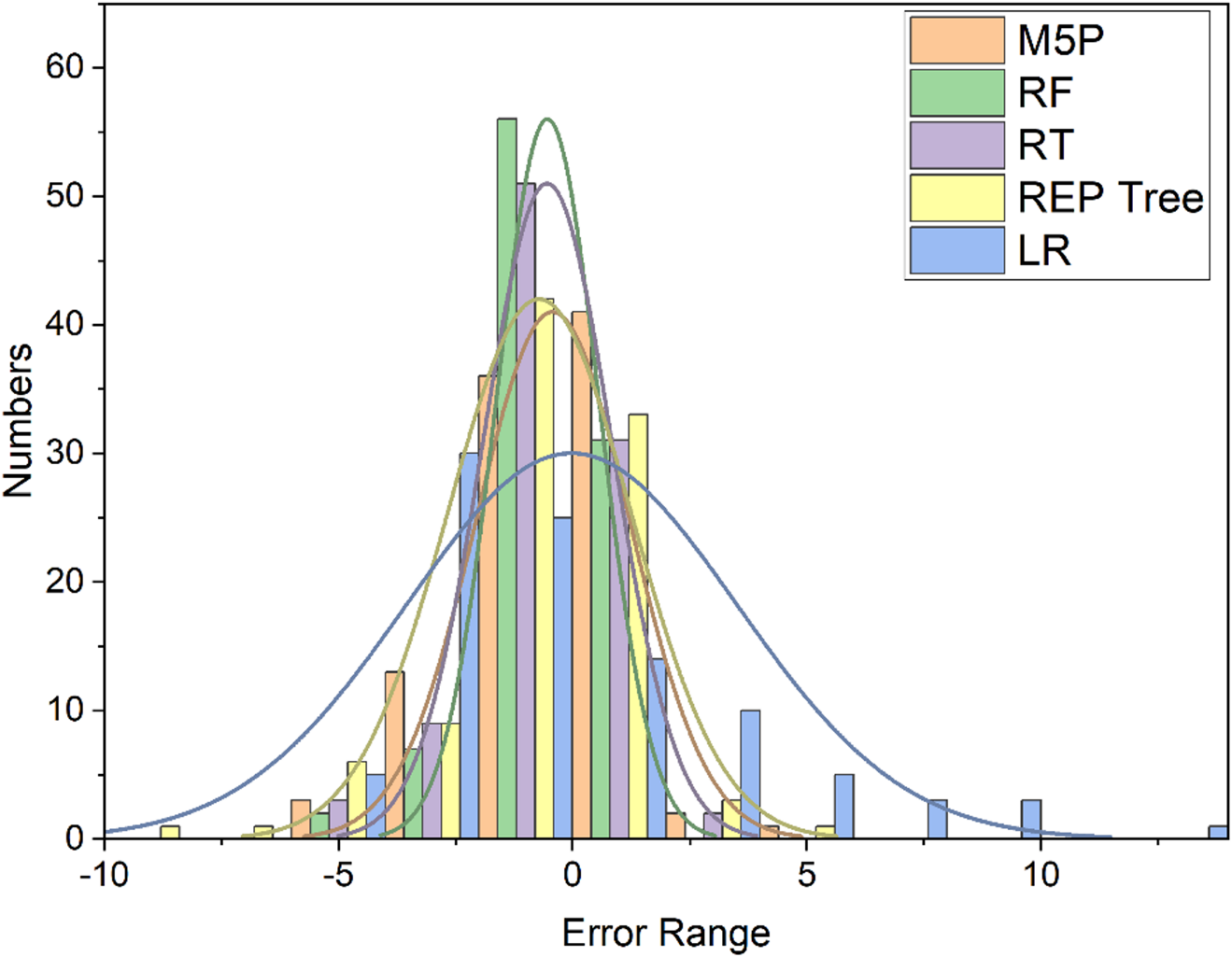
Testing-set error histograms with kernel-density overlays for LR, M5P, RT, REP-Tree, and RF models.

**Fig. 14:**
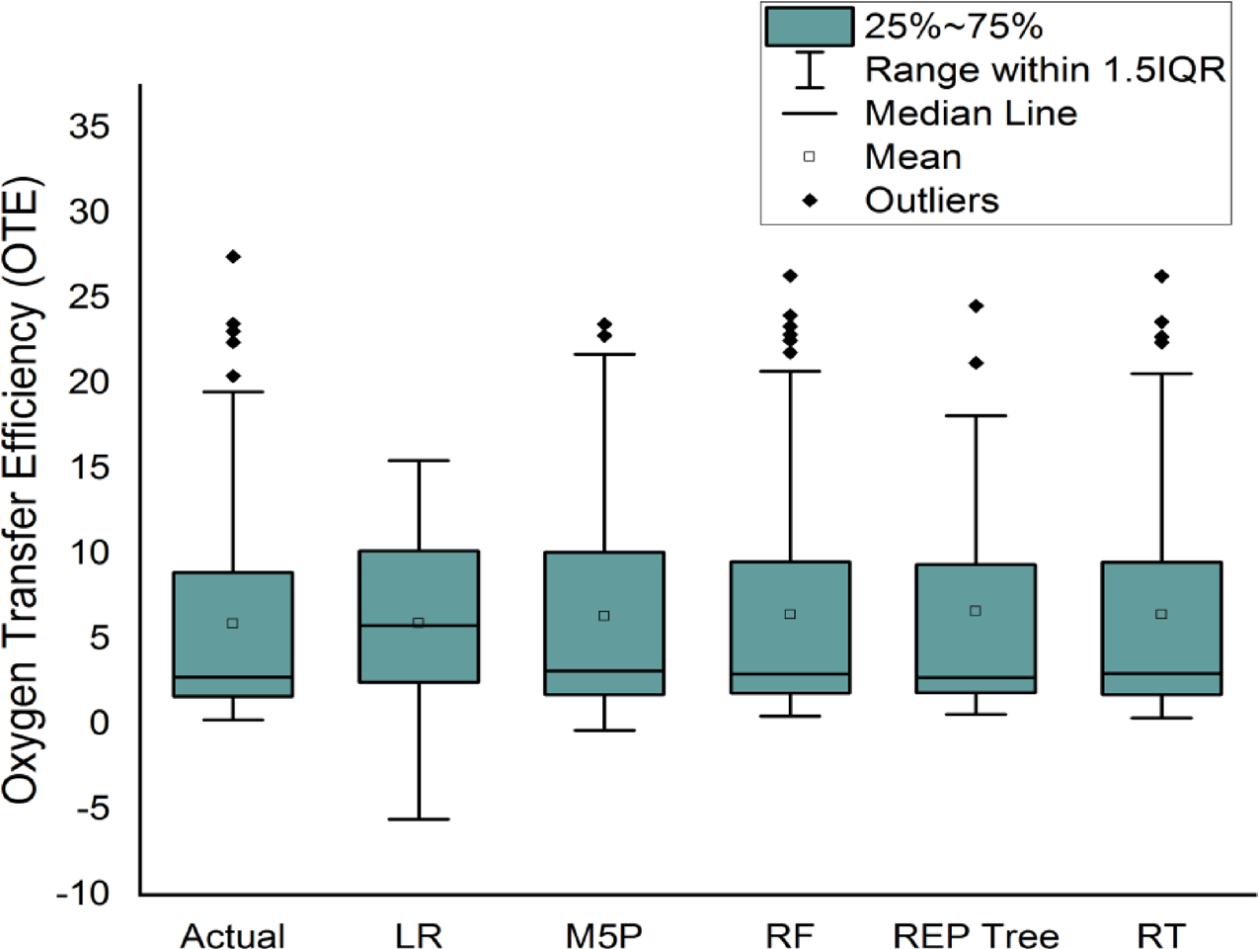
Box plot comparing the actual and applied models using the testing stage.

After evaluating all models using concordance, efficiency, and error metrics (Table 5; Fig. 12), tree-based learners surpassed the linear baseline: RF and RT track observed OTE most closely, REP-Tree and M5P are intermediate, and LR is weakest. Although RT shows a slight edge in training metrics, this does not persist on the test set, where RF achieves lower errors and higher accuracy, indicating stronger generalization and identifying RF as the most robust, practically reliable model overall. Testing-set residual histograms with kernel-density overlays (Fig. 13) reveal compact, slightly negative-centered errors for the tree-based models—signaling mild under-prediction with low variance—whereas LR exhibits a broader, heavy-tailed distribution consistent with its higher RMSE/MAE. Among these, RF and RT show the narrowest spreads, reflecting lower random scatter; RF shows the least spread overall. Distributional comparisons and descriptive statistics (Fig. 14; Table 6) further corroborate RF’s close alignment with the observed range and quartiles.

**Table 5:** Evaluation metrics for all models employed in the study.

Models	CC	MAE	RMSE	SI	NSE
	Training Data Set				
*LR*	0.8052	2.6020	3.5506	0.6676	0.6483
*M5P*	0.9685	0.8928	1.5248	0.2867	0.9351
*RF*	0.9985	0.2245	0.3380	0.0635	0.9968
*REP Tree*	0.9823	0.6596	1.1219	0.2110	0.9649
*RT*	0.9999	0.0598	0.0978	0.0184	0.9997
	**Testing Data Set**				
*LR*	0.8376	2.7532	3.5201	0.5956	0.6953
*M5P*	0.9678	1.0610	1.6804	0.2843	0.9306
*RF*	0.9894	0.7276	1.2187	0.2062	0.9635
*REP Tree*	0.9641	1.2748	2.0685	0.3500	0.8948
*RT*	0.9861	0.9282	1.4687	0.2485	0.9470

**Table 6:** Descriptive statistics for comparing actual data with applied models during the testing stage.

Statistic	Actual	LR	M5P	RF	REP Tree	RT
*Minimum*	0.2500	-5.5770	-0.3600	0.4670	0.5840	0.3700
*Maximum*	27.4500	15.4680	23.4790	26.3270	24.5530	30.9500
*1st Quartile*	1.6575	2.4890	1.7678	1.8555	1.8550	1.7513
*Mean*	5.9099	5.9278	6.3328	6.4465	6.6205	6.4506
*3rd Quartile*	8.9075	10.1623	10.0010	9.4685	9.3690	9.5200
*IQR*	7.2500	7.6733	8.2333	7.6130	7.5140	7.7688

**Fig. 15:**
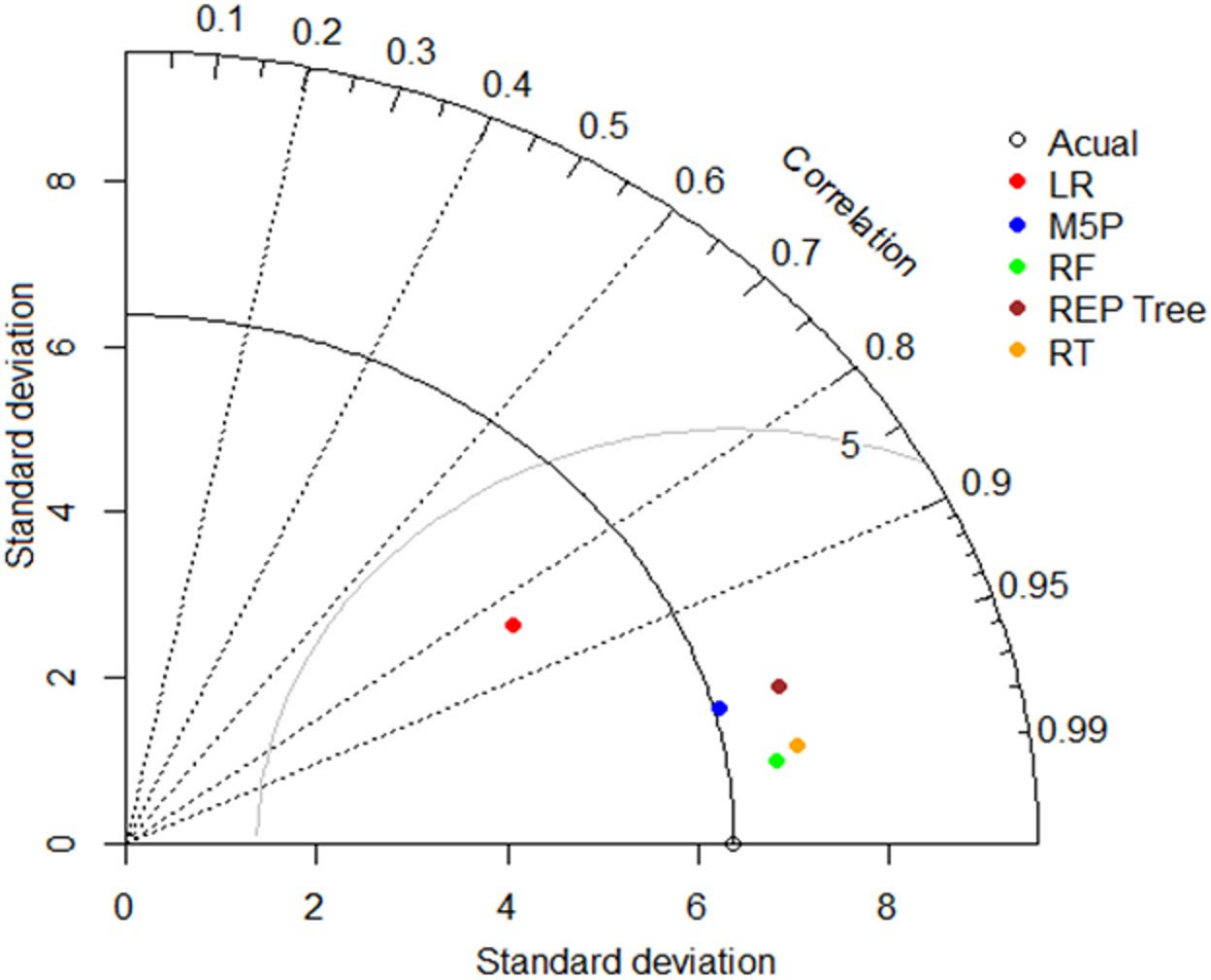
Taylor’s diagram showcasing observed and predicted OTE values across testing datasets using different models.

A Taylor diagram (Fig. 15) summarizes correlation, centered RMSE, and variability, placing RF closest to the observed reference and LR farthest—consistent with Tables 5 and 6. Complementing this, Table 7 reports residual uncertainty (mean error, SD, U95 with bounds): RF and RT yield the tightest envelopes, M5P and REP-Tree are moderately wider, and LR is widest.

**Table 7:** Residual-uncertainty metrics on the testing set: mean error, SD, and U95 with upper/lower bounds.

Models	Mean error	Std Dev error	U95	Upper bound	Lower Bound
*M5P*	-0.4229	1.6349	*±* 1.3192	7.2291	4.5907
*RF*	-0.5366	1.1000	*±* 1.2987	7.2086	4.6112
*RT*	-0.5407	1.3727	*±* 1.3090	7.2189	4.6009
*REP Tree*	-0.7106	1.9528	*±* 1.3410	7.2509	4.5689
*LR*	-0.0179	3.5385	*±* 1.4571	7.3670	4.4528

To assess whether differences are statistically meaningful, we performed paired t-tests on absolute prediction errors from the testing dataset (Table 8). RF exhibited significantly lower errors than all other models (*p* < 0.01 across comparisons), with medium–large effect sizes (|d| ≈ 0.30–0.91). M5P did not differ significantly from RT or REP Tree (*p* > 0.05). These results substantiate RF’s superiority in generalization performance.

**Table 8:** Paired t-test analysis of model performance based on absolute prediction errors over the testing dataset.

Model A	Model B	Mean Diff	95% CI Lower	95% CI Upper	t	df	*p*-value	Cohen’s d
M5P	RF	0.3334	0.1206	0.5461	3.0715	95	0.00278	0.313
M5P	RT	0.1328	-0.098	0.3636	1.1275	95	0.26237	0.115
M5P	REP Tree	-0.2138	-0.4724	0.0448	-1.6205	95	0.10844	-0.165
M5P	LR	-1.6922	-2.1175	-1.2669	-7.7989	95	0	-0.796
RF	RT	-0.2006	-0.336	-0.0652	-2.9036	95	0.00459	-0.296
RF	REP Tree	-0.5472	-0.739	-0.3554	-5.5919	95	0	-0.571
RF	LR	-2.0256	-2.4716	-1.5796	-8.9023	95	0	-0.909
RT	REP Tree	-0.3466	-0.5464	-0.1468	-3.3995	95	0.00099	-0.347
RT	LR	-1.825	-2.2245	-1.4254	-8.9529	95	0	-0.914
REP Tree	LR	-1.4784	-1.9449	-1.0119	-6.211	95	0	-0.634

**Fig. 16:**
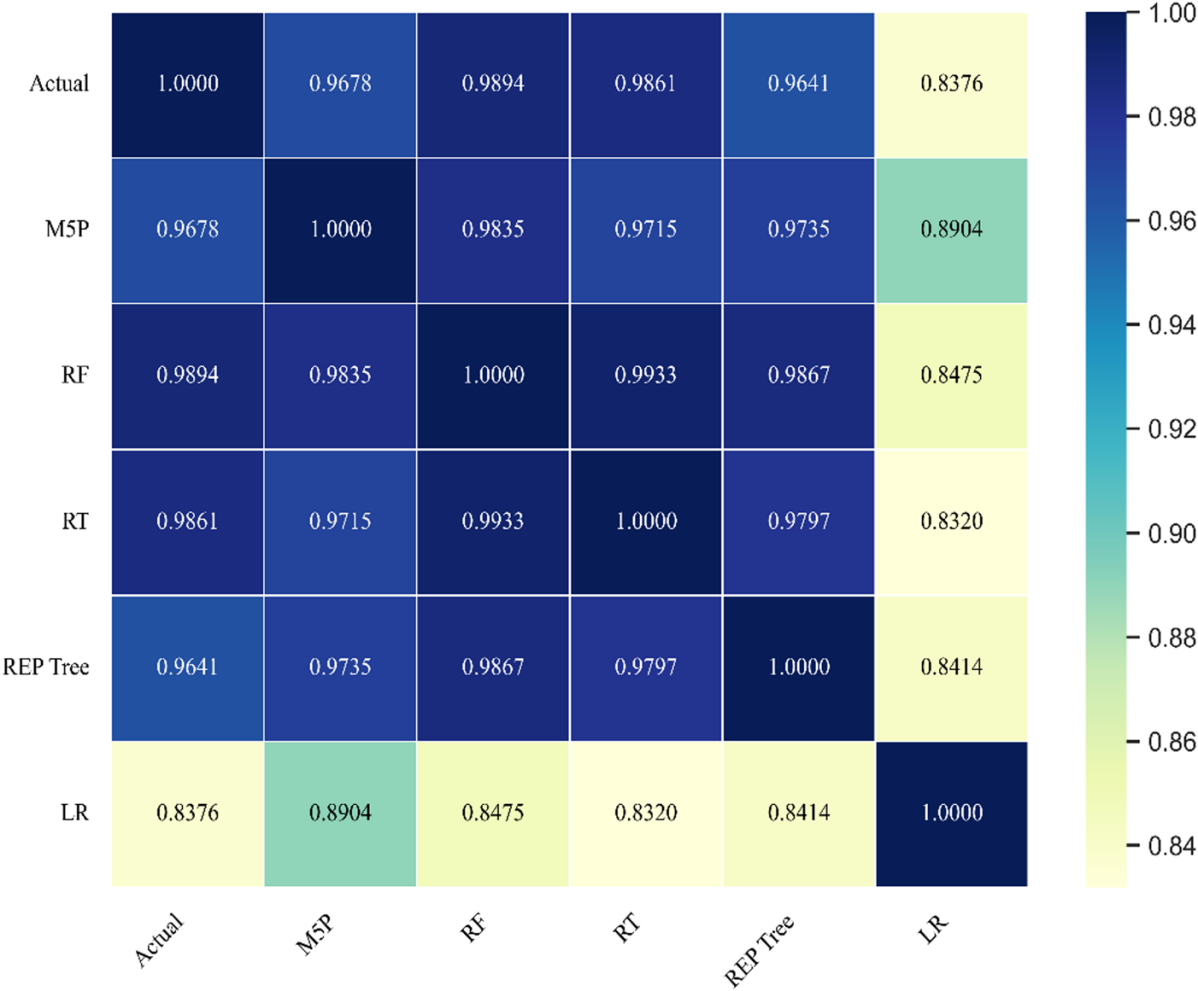
Spearman correlation heatmap showing associations between measured OTE values and predictions from all applied models suing testing dataset.

A Spearman correlation heatmap (Fig. 16) shows the strongest association with observations for RF (ρ = 0.9894), followed by RT (ρ = 0.9861) and M5P (ρ = 0.9678); LR (ρ = 0.8376) is notably lower. High inter-model correlations among tree-based methods (e.g., RF–RT) indicate similar prediction tendencies, but RF maintains the highest concordance with measured OTE, reinforcing its selection as the preferred model.

Overall, RT’s marginal training advantage does not persist under testing, whereas RF remains superior on the test set and in paired t-tests, indicating better generalization and practical reliability.

### Sensitivity analysis

A sensitivity analysis was performed using the Random Forest model to identify the most critical input variables affecting the oxygen transfer efficiency (OTE) of solid jet aerators with circular apertures. The influence of many factors, including the quantity of apertures, length of the jet, area of disc flow, and rate of discharge, were assessed in relation to their effect on OTE. Testing datasets were systematically generated by excluding individual variables one at a time, as demonstrated in Table 9. The influence of each input factor on the output variable (OTE) was assessed using CC, MAE and RMSE. Results from Table 9 indicate that discharge rate (Q) is the most significant parameter influencing the oxygen transfer efficiency of solid jet aerators with circular openings based on the dataset.

**Table 9:**
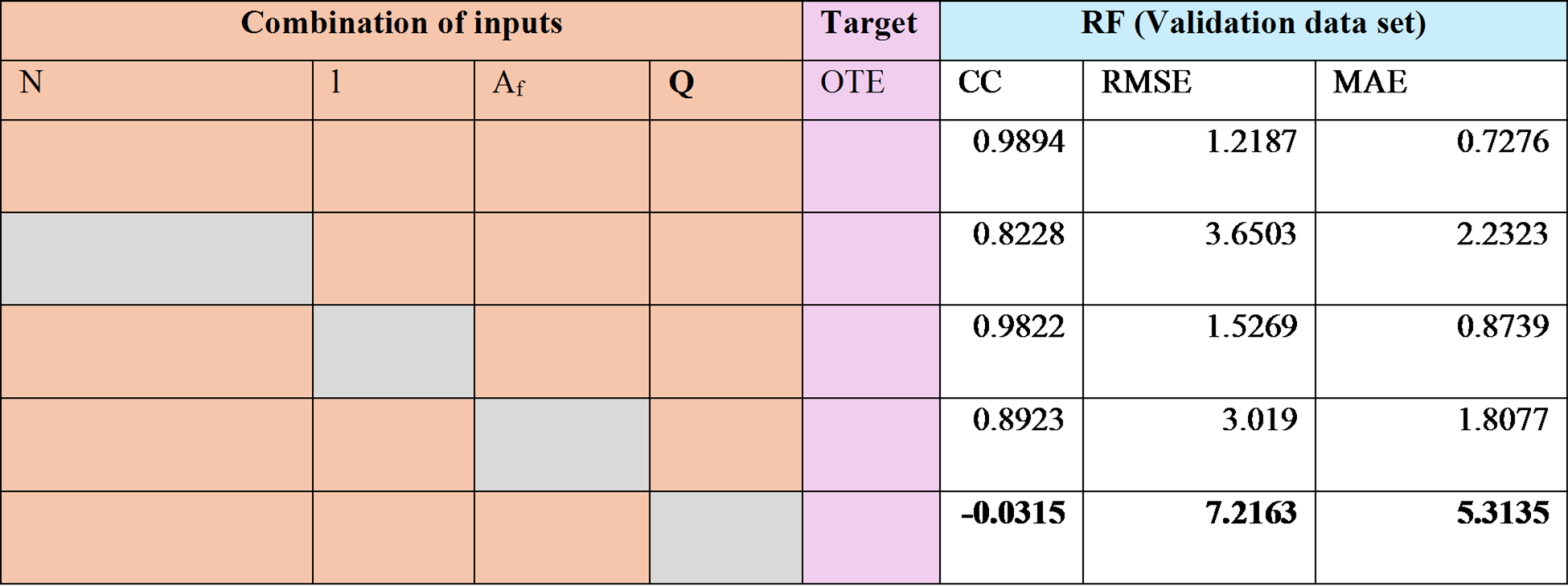
Sensitivity analysis utilising RF based model for the testing stage.

*Bold values show most significant input variable. **Grey box shows the relevant column element is missing from the data.

## Conclusion

The present study comprehensively evaluated the oxygen-transfer efficiency (OTE) of solid-jet aerators with circular apertures under varying configurations of jet number, jet length, disc flow area, and discharge rate. Several machine learning models—Linear Regression (LR), M5P, Random Tree (RT), Reduced Error Pruning (REP) Tree, and Random Forest (RF)—were applied to predict OTE, spanning baseline regression, interpretable model trees, and ensembles for a balanced comparison. Although RT showed a slight edge during training, RF consistently attained higher accuracy and lower errors on the test set, indicating stronger generalization; this ranking is corroborated by residual histograms and uncertainty statistics (U95), as well as the Taylor diagram. Sensitivity analysis identified discharge (Q) as the most influential input. Overall, advanced ML techniques—particularly RF—accurately model oxygen-transfer processes in aeration systems, with practical implications for optimizing aerator design and improving operational efficiency in wastewater and hazardous-waste treatment. These findings support sustainable water-treatment practices aligned with global environmental goals.

## Data Availability

The datasets used and/or analysed during the current study available from the corresponding author on reasonable request.

## References

[1] Mohamed, N. A., ELfarash, A., Abdel-Sater, M. A. & Hassan, E. A. Fungal diversity and composition in river nile water polluted with Chlorpyrifos insecticide. *J. Appl. Mol. Biol.* **2**, 31–51 (2024).

[2] Imtiyaz, I., Krishnakant, Shukla, B. K., Varadharajan, S. & Bharti, G. A comprehensive review of mass transfer phenomenon in gas-liquid phase flow during aeration in wastewater treatment. *Lect Notes Civ. Eng.* **281**, 127–135 (2023).

[3] Shukla, B. K., Goel, A., Sharma, P. K. & Sihag, P. Approximation of Oxygen Transfer Efficiency of Solid Jet Aerator Having Circular Opening with Kernel Function-Based Models and Random Forest Models. *Iran. J. Sci. Technol. Trans. Civ. Eng.*10.1007/s40996-025-01836-z (2025a).

[4] Novak, R. G. Techniques and factors involved in aerator selection and evaluation. *J. Water Pollut Control Fed.* **452**, 452–463 (1968).

[5] Kalinske, A. Economic evaluation of aerator systems. *Environ. Sci. Technol.* **3**, 229–234 (1969).

[6] Chanson, H. & Brattberg, T. Air entrainment by two-dimensional plunging jets: the impingement region and the very-near flow field. *Am. Soc. Mech. Eng. Fluids Eng. Div. Publ FED.* **2**, 1-8 (1998).

[7] Kalinske, A. A. Power consumption for oxygenation and mixing. *Air Water Pollut Annu. Rep.* **5**, 157 (1961). 13961939

[8] Puri, D., Sihag, P., Sadeghifar, T., Dursun, O. F. & Thakur, M. S. Soft computing-based model development for estimating the aeration efficiency through parshall flume and venturi flumes. *Multiscale Multidiscip Model. Exp. Des.* **6**, 401–413 (2023).

[9] Shukla, B. K. & Goel, A. Study on oxygen transfer by solid jet aerator with multiple openings. *Eng. Sci. Technol. Int. J.* **21**, 255–260 (2018).

[10] Shukla, B. K., Goel, A., Sharma, P. K. & Sihag, P. Advanced kernel function-based models and soft computing techniques for optimizing oxygen transfer efficiency in solid jet aerators with circular openings: a comparative study of Gaussian process regression, random forest and support vector machine approaches. *Multiscale Multidiscip Model. Exp. Des.* **8**, 108 (2025b).

[11] Tojo, K. & Miyanami, K. Oxygen transfer in jet mixers. *Chem. Eng. J.* **24**, 89–97 (1982).

[12] Deswal, S. & Verma, D. V. S. Performance evaluation and modeling of a conical plunging jet aerator. *Int. J. Aerosp. Mech. Eng.* **1**, 616–620 (2007).

[13] Pillai, N. N., Wheeler, W. C. & Prince, R. P. Design and operation of an extended aeration plant. *J. Water Pollut Control Fed.* **43**, 1484–1498 (1971). 5568367

[14] Pasrija, H. D. & Pillai, N. N. Air aspirators for oxygenation of wastewaters. *J. Inst. Public. Health Eng. India*. **3**, 7–13 (1976).

[15] Ding, W., Liu, Z. & Wang, X. Modeling of wastewater treatment and dynamic optimization control of dissolved oxygen concentration in aeration tanks. *J. Environ. Eng.* **151**, 04025050 (2025).

[16] Voipan, D., Voipan, A. E. & Barbu, M. Hybrid explainable AI framework for predictive maintenance of aeration systems in wastewater treatment plants. *Water* **17**, 2636 (2025).

[17] Saha, N., Heim, R., Mazumdar, A., Banerjee, G. & Sarkar, O. Optimization of cascade aeration characteristics and predicting aeration efficiency with machine learning model in multistage filtration. *Environ. Model. Assess.* **29**, 1079–1093 (2024).

[18] Muloiwa, M., Dinka, M. O. & Nyende-Byakika, S. Modelling and optimizing hydraulic retention time in the biological aeration unit: application of artificial neural network and particle swarm optimization. *S Afr. J. Chem. Eng.* **48**, 292–305 (2024).

[19] Quinlan, J. R. Learning with continuous classes. In *Proc. 5th Aust. Jt. Conf. Artif. Intell.* 343–348 (1992).

[20] Tiwari, N. K. & Panwar, D. Optimising venturi flume oxygen transfer efficiency using uncertainty-aware decision trees. *Water Sci. Technol.* **90**, 3210–3240 (2024). 39733451 10.2166/wst.2024.393

[21] Breiman, L. Random forests. *Mach. Learn.* **45**, 5–32 (2001).

[22] Chen, C., Liaw, A. & Breiman, L. Using random forest to learn imbalanced data. *Univ. Calif. Berkeley Stat. Dep*. **110**, 1–12 (2004).

[23] Witten, I. H., Frank, E., Hall, M. A., Pal, C. J. & Data, M. Practical machine learning tools and techniques. In *Data Mining* **2**, 403–413 (Elsevier, 2005).

[24] Sürer, Ö., Apley, D. W. & Malthouse, E. C. Coefficient tree regression: fast, accurate and interpretable predictive modeling. *Mach. Learn.* **113**, 4723–4759 (2024).

[25] Hai, T. et al. Surface water quality index forecasting using multivariate complementing approach reinforced with locally weighted linear regression model. *Environ. Sci. Pollut Res.* **31**, 32382–32406 (2024). 10.1007/s11356-024-33027-0 38653893

[26] Cheng, Q. et al. AI-Optimised aeration control in SBR systems: an inverse SVM framework toward carbon-neutral wastewater treatment. *Environ. Technol.*10.1080/09593330.2025.2562373 (2025). 41045551 10.1080/09593330.2025.2562373

[27] Kumar, M., Tiwari, N. K. & Ranjan, S. Soft computing based predictive modelling of oxygen transfer performance of plunging Hollow jets. *ISH J. Hydraul Eng.* **28**, 223–233 (2022).

[28] Dikmen, F. et al. AI-driven wastewater management through comparative analysis of feature selection techniques and predictive models. *Sci. Rep.* **15**, 25347 (2025). 40659650 10.1038/s41598-025-07124-0 PMC12259835

[29] Breiman, L. Bagging predictors. *Mach. Learn.* **24**, 123–140 (1996).

[30] Wang, H. C. et al. Multimodal machine learning guides low carbon aeration strategies in urban wastewater treatment. *Engineering* **36**, 51–62 (2024).

[31] Baylar, A., Emiroglu, M. E. & Ozturk, M. The development of aeration performance with different typed nozzles in a vertical plunging water jet system. *Int. J. Sci. Technol.* **1**, 51–63 (2006).

[32] Deswal, S. & Verma, D. V. S. Air-water oxygen transfer with multiple plunging jets. *Water Qual. Res. J.* **42**, 295–302 (2007).

[33] Hinojosa, J. et al. Determining the primary sources of fecal pollution using microbial source tracking assays combined with land-use information in the Edwards aquifer. *Water Res.* **184**, 116211 (2020). 32721766 10.1016/j.watres.2020.116211

[34] Daniil, E. I. & Gulliver, J. S. Temperature dependence of liquid film coefficient for gas transfer. *J. Environ. Eng.* **114**, 1224–1229 (1988).

[35] Breiman, L. Using adaptive bagging to debias regressions. *Tech. Rep. Stat. Dep Univ. Calif. Berkeley*. **547**, 1–16 (1999).

[36] Quinlan, J. R. Simplifying decision trees. *Int. J. Man-Mach Stud.* **27**, 221–234 (1987).

[37] Hamoud, A., Hashim, A. S. & Awadh, W. A. Predicting student performance in higher education institutions using decision tree analysis. *Int. J. Interact. Multimed Artif. Intell.* **5**, 26–31 (2018).

[38] Cutler, D. R. et al. Random forests for classification in ecology. *Ecology* **88**, 2783–2792 (2007). 18051647 10.1890/07-0539.1

[39] Barddal, J. P., Gomes, H. M., Enembreck, F. & Pfahringer, B. A survey on feature drift adaptation: Definition, benchmark, challenges and future directions. *J. Syst. Softw.* **127**, 278–294 (2017).

[40] Kashi, H., Emamgholizadeh, S. & Ghorbani, H. Estimation of soil infiltration and cation exchange capacity based on multiple regression, ANN (RBF, MLP), and ANFIS models. *Commun. Soil. Sci. Plant. Anal.* **45**, 1195–1213 (2014).

